# The Kaposi's sarcoma-associated herpesvirus (KSHV) non-structural membrane protein K15 is required for viral lytic replication and may represent a therapeutic target

**DOI:** 10.1371/journal.ppat.1006639

**Published:** 2017-09-22

**Authors:** Bizunesh Abere, Tamrat M. Mamo, Silke Hartmann, Naira Samarina, Elias Hage, Jessica Rückert, Sven-Kevin Hotop, Guntram Büsche, Thomas F. Schulz

**Affiliations:** 1 Institute of Virology, Hannover Medical School, Hannover, Germany; 2 German Centre for Infection Research, Hannover–Braunschweig Site, Germany; 3 Institute of Molecular Biology, Hannover Medical School, Hannover, Germany; 4 Department of Chemical Biology, Helmholtz Center for Infection Research, Braunschweig, Germany; 5 Institute of Pathology, Hannover Medical School, Hannover, Germany; University of North Carolina at Chapel Hill, UNITED STATES

## Abstract

Kaposi’s sarcoma-associated herpesvirus (KSHV) is the infectious cause of the highly vascularized tumor Kaposi’s sarcoma (KS), which is characterized by proliferating spindle cells of endothelial origin, extensive neo-angiogenesis and inflammatory infiltrates. The KSHV K15 protein contributes to the angiogenic and invasive properties of KSHV-infected endothelial cells. Here, we asked whether K15 could also play a role in KSHV lytic replication. Deletion of the K15 gene from the viral genome or its depletion by siRNA lead to reduced virus reactivation, as evidenced by the decreased expression levels of KSHV lytic proteins RTA, K-bZIP, ORF 45 and K8.1 as well as reduced release of infectious virus. Similar results were found for a K1 deletion virus. Deleting either K15 or K1 from the viral genome also compromised the ability of KSHV to activate PLCγ1, Erk1/2 and Akt1. In infected primary lymphatic endothelial (LEC-rKSHV) cells, which have previously been shown to spontaneously display a viral lytic transcription pattern, transfection of siRNA against K15, but not K1, abolished viral lytic replication as well as KSHV-induced spindle cell formation. Using a newly generated monoclonal antibody to K15, we found an abundant K15 protein expression in KS tumor biopsies obtained from HIV positive patients, emphasizing the physiological relevance of our findings. Finally, we used a dominant negative inhibitor of the K15-PLCγ1 interaction to establish proof of principle that pharmacological intervention with K15-dependent pathways may represent a novel approach to block KSHV reactivation and thereby its pathogenesis.

## Author summary

Both the latent and lytic replication phases of the KSHV life cycle are thought to contribute to its persistence and pathogenesis. The non-structural signaling membrane protein K15 is involved in the angiogenic and invasive properties of KSHV-infected endothelial cells. Here we show that the K15 protein is required for virus replication, early viral gene expression and virus production through its activation of the cellular signaling pathways PLCγ1 and Erk 1/2. K15 is abundantly expressed in KSHV-infected lymphatic endothelial cells (LECs) and contributes to KSHV-induced endothelial spindle cell formation. The abundant K15 protein expression observed in LECs is also observed in KS tumors. We also show that it may be possible to target K15 in order to intervene therapeutically with KSHV lytic replication and pathogenesis.

## Introduction

Kaposi’s sarcoma-associated herpesvirus (KSHV), also known as human herpesvirus –8 (HHV-8), causes Kaposi’s sarcoma (KS) [1] and two lymphoproliferative disorders: primary effusion lymphoma (PEL) [2] and the plasmablastic variant of multicentric Castleman’s disease (MCD) [3]. KS is the commonest neoplasm associated with KSHV infection and among the first clinical manifestations in untreated AIDS patients [4]. Histologically, it is characterized by KSHV latent nuclear antigen (LANA) positive proliferating spindle cells of endothelial origin, infiltrates of immune cells such as monocytes, plasma cells, and B and T cells, as well as extensive neo-angiogenesis with slit-like vascular spaces which allows the extravasation of erythrocytes to the surrounding tissue and results in the characteristic purplish appearance of the lesion [5]. In the later nodular stage of KS the majority of spindle cells, 90%, harbor the virus in its latent state, while a small proportion of these cells also display lytic replication, an important phenomenon in the pathogenesis of KS [6–8]. Cells supporting KSHV lytic replication are thought to contribute to the progression of KS through secretion of proangiogenic and proinflammatory factors, which can act both in an autocrine and paracrine manner and can release infectious virus that replenishes the pool of infected cells recruited to latency [5, 7, 9, 10].

The environmental and physiological cues leading to KSHV lytic reactivation in infected individuals are not well defined. However, inflammation [11, 12], hypoxia [13–15], oxidative stress [16] and co-infection with other viruses such as HIV-1[17–19] and other herpesviruses [20–22] have been shown, mostly in experimental settings, to trigger KSHV reactivation from latency through the release of inflammatory cytokines such as IFN-γ, hepatocyte growth factor/scatter factor and Oncostatin M [11, 12, 17, 23, 24] or the production of reactive oxygen species (ROS) [16, 25, 26]. These stimuli induce KSHV reactivation through the activation of specific intracellular signaling pathways and their downstream transcription factors such as AP-1 which can directly act on the promoters of several viral lytic genes including the master lytic switch protein RTA (replication and transcription activator), which is sufficient to disrupt KSHV latency [27, 28].

The activation of all three mitogen-activated protein kinase (MAPK) pathways, extracellular signal-regulated kinase (ERK), c-Jun N-terminal kinase (JNK) and p38, as well as their downstream transcription factors AP-1 and Ets-1 have been shown to occur as a result of ROS production from oxidative stress and inflammation, during co-infection with other viruses or treatment with chemical inducers such as 12-*O*-tetradecanoyl-phrobol-13-acetate (TPA) and to be crucial for KSHV reactivation from latency as well as viral infection and replication during primary infection of endothelial cells [25, 29–35]. Increased expression of Pim-1 and Pim-3 kinases in response to cytokines or chemical inducers of KSHV reactivation can lead to the phosphorylation of the latency-associated nuclear antigen (LANA) on serine residues 205 and 206 which counteracts its repressive function on viral lytic transcription [36]. Blocking the activation of protein kinase C (PKC) isoforms by using GF 109203X or the PKCδ isoform specifically decreases TPA-induced KSHV reactivation [37]; while intracellular calcium mobilization elicits KSHV lytic replication through the activation of the Ca^++^ dependent calcineurin-NFAT pathway independently of PKC activation [38]. In addition to the ERK MAPK pathway, co-infection of BCBL-1 cells with either HIV-1 or HSV-1 activates the phosphatidylinositol 3-kinase (PI3K)-Akt pathway, inactivates the downstream GSK-3β through its phosphorylation and decreases the expression of the negative regulator PTEN which in turn resulted in KSHV reactivation from latency [34, 35, 39]; on the other hand, pharmacological inhibition of the PI3K-AKT pathway induced KSHV lytic reactivation in BC-3 cells [40]. Moreover, activation of the toll-like receptors TLR7/8, crucial players in pathogen recognition and activation of the host innate immune response, using either its agonists or as a result of co-infection with vesicular stomatitis virus (VSV) have also been shown to be crucial for KSHV lytic gene transcription and replication [41].

The open reading frame (ORF) K15, located at one end of the long unique coding region of the KSHV genome between ORF 75 and the terminal repeat region, contains eight alternatively spliced exons encoding multiply spliced variants of the K15 transcript [42–44]. These transcripts encode a K15 protein containing a variable number of membrane spanning domains with an attached short amino (N)- terminal and long carboxyl (C)-terminal cytoplasmic domain; the longest transcript (containing all eight exons) codes for a protein of 45 KDa apparent molecular weight with 12 predicted membrane-spanning domains [44, 45]. So far, at least three highly divergent (less than 33% amino acid sequence similarity), K15 alleles have been identified in different KSHV genomes, designated as predominant (P), minor (M) and N [43–46]. Despite their low sequence similarity, all three K15 alleles feature several conserved putative signaling motifs such as a proline-rich Src homology 3 (SH3)-binding site (PPLP) and two SH2-binding sites (YASIL and YEEVL) in their C-terminal cytoplasmic tail [43, 44]. Upon ectopic expression, K15 recruits and constitutively activates the phospholipase C γ1 (PLCγ1) protein by using a constitutively phosphorylated tyrosine residue at its second SH2-binding site (Y^481^EEVL) and one of two SH2 domains of PLCγ1 [42, 45, 47–49]. K15 also induces the activation of the two MAP-kinases Erk2 and JNK, calcineurin-NFAT as well as NF-κB pathways leading to the activation of the NFAT and AP-1 transcription factors and expression of proangiogenic and proinflammatory factors such as Dscr-1, interleukin (IL)-6, IL-8, IL-1α/β, CCL20, CCL2, CXCl3 and Cox-2 as well as intracellular calcium ion (Ca^++^) mobilization [42, 45, 47–50]. Depletion of K15 from the infected cell or its deletion from the viral genome reduces the invasiveness of KSHV infected endothelial cells and their ability to form capillary tubes in a PLCγ1 dependent manner [48, 49], linking its activation of PLCγ1-dependent signaling pathways to KSHV induced angiogenesis. K15 has also been shown to interact with the anti-apoptotic protein HAX1 and induce the expression of a number of anti-apoptotic genes including *birc2*, *brc3*, *bf*, *A20* and *bcl2a1* which can provide a survival advantage to the infected cell [47, 51, 52].

Given the importance of inflammatory and angiogenic factors as well as intracellular signaling pathways such as the MAPK pathways in KSHV lytic replication (discussed above), we investigated whether K15 could also play a role in this regard. Our results revealed that the expression of the K15 protein in the infected cell is crucial for KSHV lytic replication and its reactivation from latency; K15 also contributes to the activation of PLCγ1, Erk1/2 and Akt1 signaling during viral lytic replication. We establish K15 as one of the viral proteins responsible for KSHV induced spindle cell formation in stably infected lymphatic endothelial cells (LECs) and show its abundant expression at the protein level in KS biopsies. As a proof of principle, we used a dominant negative inhibitor to intervene with the K15-PLCγ1 interaction, K15-dependent signaling pathways and KSHV lytic replication, suggesting that the recruitment of PLCγ1 to K15 might represent a druggable target to block KSHV lytic gene expression at an early stage in its replication cycle and thereby KSHV pathogenesis.

## Results

### The absence of K15 from KSHV infected cells compromises the ability of the virus to reactivate from latency

**Fig 1:**
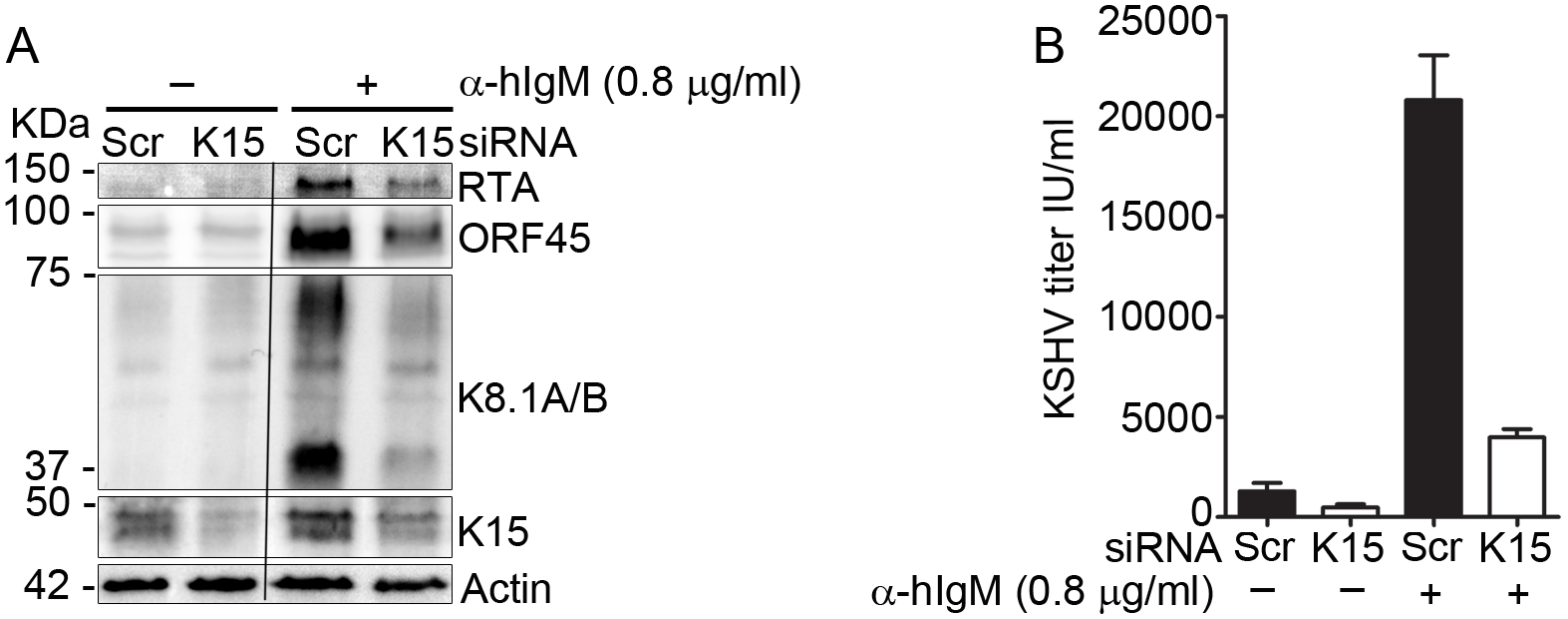
Depleting the K15 mRNA from KSHV infected cells compromises its ability to undergo lytic reactivation. BJAB-rKSHV.219 cells were microporated with either a control siRNA (Scr) or siRNA against the KSHV K15 gene and the lytic cycle was induced 24 hours later with 0.8 μg/ml of anti-hIgM antibody. Forty-eight hours after lytic induction, **(A)** cells were lysed and the expression level of the indicated lytic viral proteins was assessed by western blot and **(B)** infectious virus titer released into the supernatant was determined by infecting HEK-293 cells. Results are representative of two or more independent experiments. Bar graphs **(B)** represent the means ± SD of three replicates.

Previously, our group has established BJAB-rKSHV cells [39, 53], a B cell line stably infected with rKSHV.219, in which the KSHV lytic cycle can be activated by cross-linking the B-cell receptor (BCR) using an antibody to IgM. This culture system obviates the need for chemical inducers or RTA overexpression and produces a relatively high amount of infectious virus in contrast to PEL cell lines and other KSHV-infected adherent cell lines. To investigate whether K15 could play a role in KSHV lytic reactivation in this system, the K15 mRNA was depleted using an siRNA targeting exon 8 of the K15 mRNA, and the KSHV lytic cycle was induced by using a sub-optimal amount of anti-human IgM antibody (0.8 μg/ml). Viral lytic replication was then assessed by western blot analysis of the expression of KSHV lytic proteins RTA, ORF 45 and K8.1 as well as infectious virus release. As shown in Fig 1A, cross-linking the BCR with anti-IgM antibody induced the expression of the indicated KSHV lytic proteins in cells treated with the control, non-targeting/scrambled (Scr) siRNA. In contrast, knock down of K15 from these cells led to reduced expression of early lytic viral proteins including the master lytic switch protein RTA as well as ORF 45, indicating that K15 expression is required at an early step during KSHV reactivation; the expression of the late lytic structural glycoprotein K8.1 was also inhibited in response to K15 depletion. An alternative explanation can also be that K15 is involved later during the lytic cycle, by inhibiting a positive feed-back loop during the activation of the lytic cycle. Further analysis of virus production from these cells revealed that knock down of K15 also strongly reduced the amount of infectious virus released after anti-IgM antibody treatment (Fig 1B). We conclude from these results that K15 plays an important role during early viral lytic gene expression and productive replication in this system.

**Fig 2:**
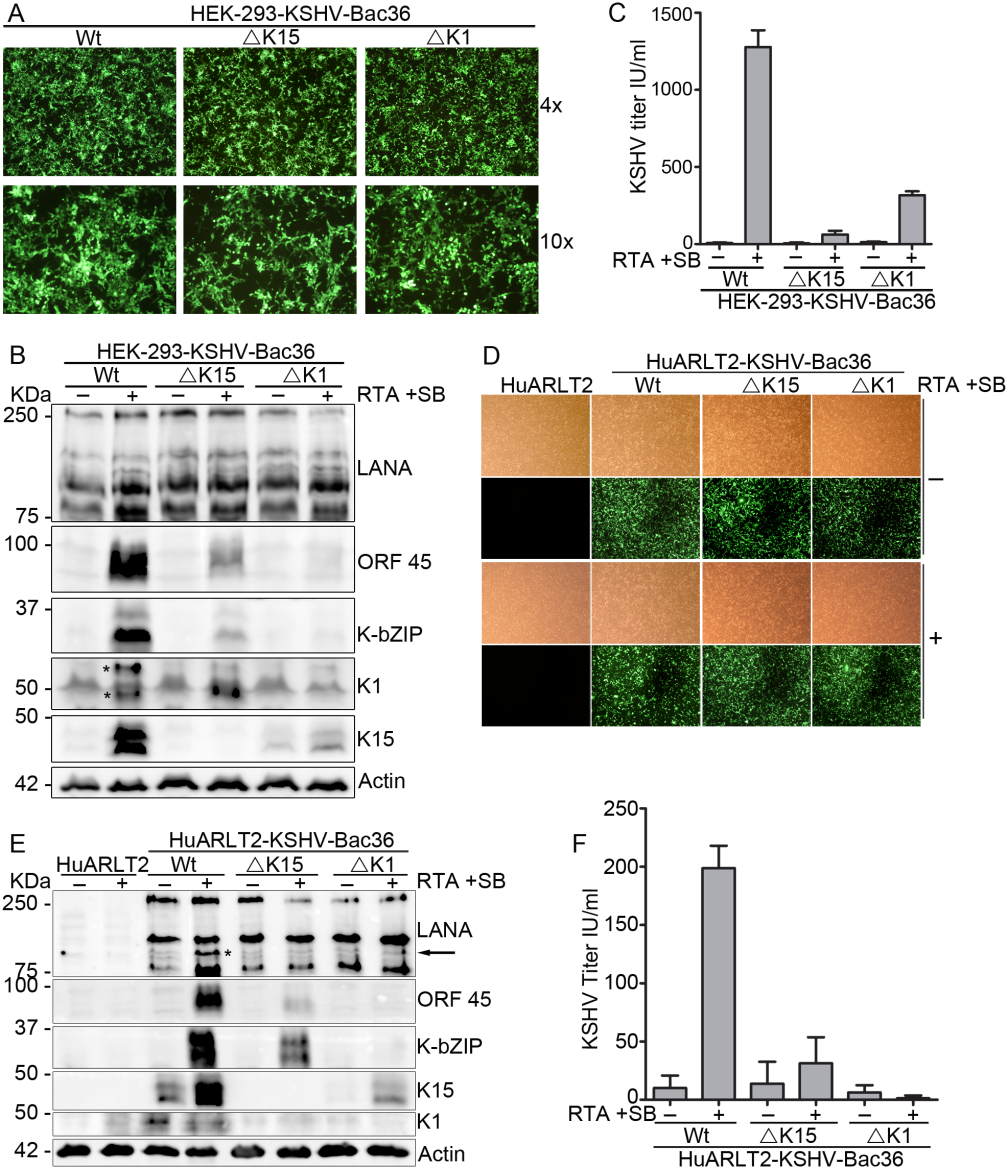
Absence of either the K1 or K15 gene from the viral genome impairs KSHV lytic reactivation. **(A)** Stably transfected polyclonal HEK-293-KSHV-Bac36 Wt/ΔK15/ΔK1 cells. In these cells, the KSHV lytic cycle was induced with 1 mM Sodium Butyrate (SB) and SF-9 cell supernatant containing KSHV-RTA expressing baculovirus. After 48 hours, cells and culture supernatant were collected; **(B)** expression level of the indicated lytic viral proteins and **(C)** infectious virus titer was analyzed as described in materials and methods. **(D)–(F)** In HuARLT2 cells stably infected with the indicated KSHV-Bac36 viruses, the KSHV lytic cycle was induced as before by using a cocktail of SB and RTA. **(D)** Stably infected cells after 48 hours with or without induction of the lytic cycle. **(E)** The expression level of the indicated lytic proteins and **(F)** infectious virus titer analyzed as described in materials and methods. Results are representative of at least three independent experiments. Bar graphs represent the means ± SD of **(C)** two or **(F)** four replications. * denotes K1 bands (panel **B**)or low molecular weight isoforms of LANA (panel **E;** see text).

To follow up our observation with another approach, we used a recombinant KSHV genome cloned in the Bacterial artificial chromosome clone 36 backbone (KSHV-Bac36) [54], which harbors a Hygromycin resistance gene as a selectable marker and expresses GFP as a marker of infection. The gene encoding the K15 protein (nucleotide sequence between 135338 and 136900) was then deleted to generate a KSHV-Bac36ΔK15 construct, as described in [48]. The other KSHV non-structural signaling membrane protein K1, encoded by the K1 gene at the opposite end of the viral genome, also activates a set of signaling pathways which can induce the expression of inflammatory and angiogenic factors similar to the K15 protein [55–58]. Recently, Zhang et al. reported that K1 is required for the activation of the KSHV lytic cycle [59]. Also Steinbrück et al. reported that the KSHV K1 and K15 proteins control a related set of cellular genes when substituted for the LMP2A gene in the EBV genome, an indication that their functions might overlap [52]. To compare the role of K1 with that of K15 during KSHV reactivation, we also produced a K1 deletion construct, KSHV-Bac36ΔK1, lacking the nucleotide sequence between nt 104 and 970. The integrity of the wild type as well as the two KSHV-Bac36 deletion mutant constructs was first validated by deep sequencing of the complete genome (S1 Fig). The increased sequence coverage of the region between ORFs K4.2 and ORF 19 is due to the duplication of this region in the TR of this Bac [60]. Stably transfected, polyclonal HEK-293 cells were established for all three constructs (Fig 2A). In these cells, the KSHV lytic cycle was induced by using a reactivation cocktail of SF-9 cell supernatant containing a baculovirus expressing the KSHV RTA, which from now on will be referred as only RTA, and sodium butyrate (SB) as described before [61]. Treatment of the HEK-293 cells stably harboring the wild type virus with the reactivation cocktail induced the expression of the KSHV immediate-early lytic genes K-bZIP and ORF 45 as well as of K1 and K15 (Fig 2B). In agreement with our K15 knock down results in the Bjab-rKSHV system above (Fig 1) and with a recent report using a K1 deletion virus [59], the absence of either the K1 or K15 gene reduced the ability of KSHV to reactivate from latency, as evidenced by the decreased expression level of KSHV early lytic proteins K-bZIP and ORF45 as well as reduced levels of infectious virus released into the supernatant of these cultures (Fig 2B and 2C).

To extend our investigation to endothelial cells, which are thought to be targets of KSHV infection *in vivo* and relevant to KS pathogenesis, HuARLT2 cells (an immortalized endothelial cell line) [62] were stably infected with the wild type or either of the deletion mutant viruses as described in materials and methods (Fig 2D), and the KSHV lytic cycle was induced using a cocktail of RTA and SB. In these endothelial cells, K15 expression is already detectable before lytic induction, as reported previously [48, 49] and treatment with the reactivation cocktail further increased its expression as well as the expression of the two early lytic marker proteins K-bZIP and ORF 45 (Fig 2E). Similar to the results described above (Figs 1 and 2), the expression of these two KSHV lytic proteins as well as virus production were both attenuated in reactivated HuARLT2 cells stably infected with the recombinant viruses lacking either the K1 or K15 gene in comparison to cells infected with the wild type virus (Fig 2E and 2F). We have recently reported [63] an increase in the expression of low molecular weight isoforms of the KSHV LANA protein upon KSHV lytic reactivation. In agreement with this previous finding, induction of the lytic cycle increased the expression level of these low molecular weight LANA isoforms in the HuARLT2-KSHV-Bac36 Wt cells, but not when either the K1 or K15 gene is lacking from the viral genome (Fig 2E). Regarding the role of K1 in KSHV reactivation, our results are consistent with the finding recently reported by Zhang et al. who used a KSHV-Bac16 construct carrying either five stop codons inside the K1 gene or a K1 deletion in HEK-293 or iSLK cells [59]. Together, these results establish a role for both the K1 and K15 proteins in KSHV lytic replication.

### Deleting the K15 gene from the viral genome impairs the activation of PLCγ1, Akt1 and Erk1/2 signaling during KSHV reactivation

**Fig 3:**
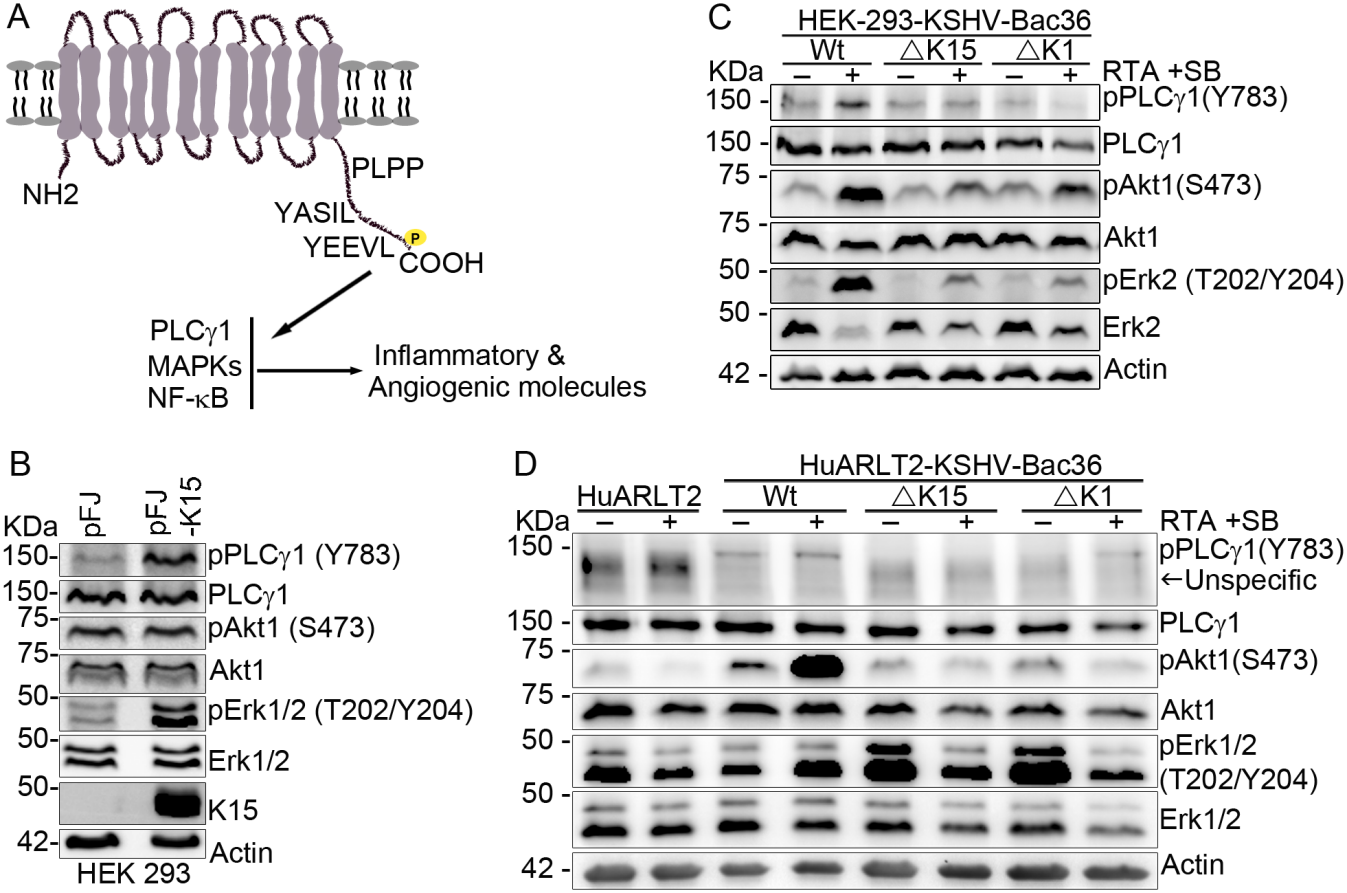
Deleting either K1 or K15 from the viral genome compromises PLCγ1, Akt1 and Erk1/2 signaling activation during KSHV reactivation. **(A)** Schematic representation of K15 activated signaling. HEK-293 cells were transfected with a plasmid DNA expressing K15 or an empty vector control; cells were collected 48 hours after transfection and **(B)** the phosphorylation level of the indicated signaling components was analyzed by western blot. The KSHV lytic cycle was induced in either **(C)** HEK-293 or **(D)** HuARLT2 cells stably infected with the indicated KSHV Bac36 viruses; forty eight hours after lytic induction, cells were collected and western blot was performed as in **B**. Results are representative of at least two independent experiments.

Upon ectopic expression, K15 has been shown to activate the PLCγ1-calcineurin-NFAT, MAP-Kinase as well as the NF-κB pathways (Fig 3A) [45, 47, 48, 50], which are crucial both during KSHV primary infection as well as during its reactivation from latency (see Introduction). As a first step in investigating the role of K15-dependent signaling pathways in KSHV lytic reactivation, we transfected HEK-293 cells with a K15 expression vector and measured the phosphorylation levels of PLCγ1(Tyr783), Akt1 (Ser473) and Erk1/2(Thr202/Tyr204) on western blots by using phospho-specific antibodies against the indicated residues. In line with experiments reported earlier [45], in which co-transfection of Erk2 together with K15 had been shown to increase Erk2 kinase activity as measured by the increased phosphorylation of the myelin basic protein (MBP) in an *in vitro* kinase assay, we found that endogenous Erk1/2 protein is phosphorylated on Thr202 and Tyr204 residues in response to K15 expression (Fig 3B). Consistent with previous results in endothelial cells from our group [48], K15 expression in HEK-293 cells also induced the activation of the PLCγ1 pathway as evidenced by its increased phosphorylation on Tyr783; in contrast, we did not observe any activation of the PI3K-Akt pathway in these cells in response to K15 expression as shown by the lack of increased Akt1 phosphorylation on Ser473 (Fig 3B). Next, we investigated the activation level of these signaling components in HEK-293 cells stably transfected with the KSHV-Bac36 wild type virus before and after the induction of the viral lytic cycle and showed that PLCγ1, Akt1 and Erk2 are activated upon lytic replication as can be seen from their increased phosphorylation at the indicated residues (Fig 3C). Deleting either K1 or K15 from the viral genome compromised the ability of KSHV to activate PLCγ1, Akt1 and Erk1/2 upon lytic reactivation in HEK-293 cells (Fig 3C). The fact that the lack of K15 in the infected cell abolished Akt1 phosphorylation in response to reactivation (Fig 3C), even though K15 did not activate this pathway directly when expressed in isolation in these cells (Fig 3B) could be due to the decreased lytic replication which results in decreased expression of other KSHV lytic proteins such as vGPCR and K1 that are known to activate the PI3K-Akt pathway directly (reviewed in [64]). In contrast, K15 activates phosphorylation of Erk1/2 and PLCγ1 directly (Fig 3B), and the decreased levels of Erk1/2 and PLCγ1 phosphorylation in cells infected with the K1 or K15 deletion viruses could therefore be the direct result of the absence or reduced expression of K15 in the case of the K1 deletion virus.

Consistent with the higher level of K15 expression in uninduced KSHV infected endothelial (HuARLT2-rKSHV) cells than in HEK-293 cells (Fig 2B and 2E), there is already a detectable level of PLCγ1 (Tyr783) phosphorylation in the uninduced KSHV-infected HuARLT2 cells compared to the uninfected control cells. PLCγ1 phosphorylation further increased upon induction of the lytic cycle, in line with the increased levels of K15 following lytic reactivation (Fig 3D). Similar to the HEK-293 cells, deleting either K1 or K15 from the viral genome also compromised the ability of KSHV to activate PLCγ1 and Akt1 (Fig 3D) upon lytic reactivation in the HuARLT2 cells. However, unlike either PLCγ1 or Akt1, the basal level of Erk1/2 phosphorylation on Thr202/Tyr204 is already abundant in the uninfected HuARLT2 cells (Fig 3D), which could be due to the hTERT and SV40 large T antigen used for immortalization of these cells (see Materials and methods). We also observed an increased Erk1/2 (Thr202/Tyr204) phosphorylation without induction of the lytic cycle in these endothelial cells infected with viruses lacking either the K1 or K15 gene which decreased upon lytic reactivation (Fig 3D).Taken together, our results so far indicate that the K1 and K15 proteins play an important role during KSHV lytic replication and its activation of cellular signaling pathways and that the absence of K1 or K15 affects the PI3K-Akt and MEK/Erk pathways in endothelial cells in a manner that would not have been predicted from the outcome of experiments involving overexpression of these proteins.

**Fig 4:**
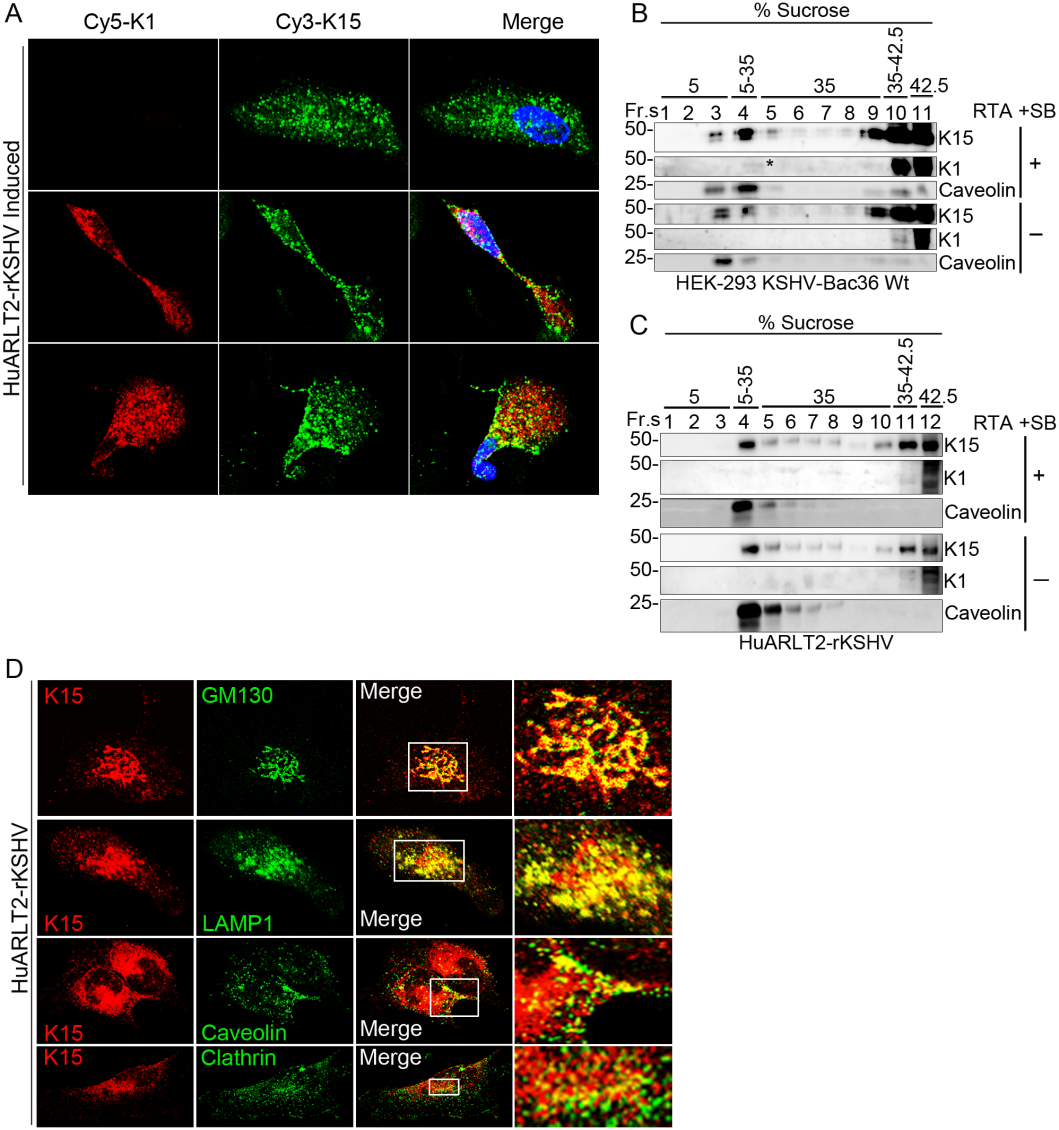
Sub-cellular localization of the K1 and K15 proteins in KSHV infected cells. **(A)** HuARLT2-rKSHV cells after lytic induction were co-stained with a rat anti-K15 and a mouse anti-K1 monoclonal antibodies followed by Cy3-conjugated anti-rat IgG (Green) and a Cy5-conjugated anti-Mouse IgG (Red) secondary antibodies; representative cells expressing only K15 (upper panel) or together with K1(lower two panels) are shown. **(B and C)** Cellular lysate from either HEK-293-KSHV-Bac36 Wt or HuARLT2-rKSHV cells with or without induction of the lytic cycle was subjected to membrane flotation assay and fractions of the sucrose gradient were analyzed for the presence of the indicated proteins by western blot. **(D)** HuARLT2-rKSHV cells without induction of the KSHV lytic cycle were co-stained with a rat anti-K15 and a rabbit antibody to the indicated cellular organelle markers followed by Cy3-conjugated anti-rat IgG (red) and a FITC-conjugated anti-rabbit IgG (green) secondary antibodies. Images **(A and D)** were acquired using a Leica confocal microscope. Experiments were performed two or more times.

Since the phenotypes of both K1 and K15 deletion mutant viruses were similar in regard to virus reactivation and signaling activation (at least for the signaling components shown here), we wondered whether the two proteins might be present in similar cellular compartments. To investigate this in the context of viral infection (in stably infected HuARLT2-rKSHV cells after lytic induction), an immunofluorescence assay (IFA) was performed using monoclonal antibodies to K1 [65] and K15 [49]; examples of cells expressing K15 (shown in green) alone or together with K1 (shown in red) are shown in Fig 4A. As can be seen in Fig 4A, K1 shows a uniform localization throughout the cytoplasm, while K15 is found in large vesicle-like structures more abundant in the perinuclear area and in the cell periphery. Cells co-expressing the two proteins show no clear co-localization (Fig 4A).

To further understand the intracellular localization of these two proteins, we analyzed their incorporation into lipid rafts. Lipid rafts are highly dynamic membrane microdomains formed by the association of glycosphingolipids with cholesterol that are involved in protein/lipid trafficking and cellular signal transduction [66, 67]. Isolation of detergent-resistant membranes (DRMs) by lysis of cells in cold nonionic detergents such as Triton X-100 coupled with ultracentrifugation in a sucrose gradient, known as flotation assay, is a commonly used method to assess the composition of lipid rafts and associated signaling proteins. In this assay, DRMs/lipid rafts float to a position of lower density, while soluble proteins remain at the bottom of the gradient with the high-density sucrose. Using this biochemical assay, we showed previously that the K15 protein expressed from a transfected plasmid associates with lipid rafts in Cos7 cells [45]. Here, we assessed the association of both K1 and K15 in KSHV-infected cells with DRMs both before and after induction of the lytic cycle. In HEK-293 cells stably infected with KSHVBac36 virus, a portion of the K15 protein can be detected in fractions 3 and 4 of the sucrose gradient in the same manner as the lipid raft marker protein Caveolin, while a significant portion still remains at the bottom of the gradient in fractions 9 to 11 which contains solubilized proteins (Fig 4B). We did not observe a differential localization of K15 to or away from lipid rafts in response to KSHV lytic reactivation in these cells (Fig 4B). Unlike K15, most of the K1 protein remained in the soluble fraction at the bottom of the sucrose gradient (fractions 10 to 11) with a very small portion of it floating to fraction 4 after induction of the lytic cycle (Fig 4B). Similar results were observed in the stably infected HuARLT2 cells (Fig 4C), although K1 was hardly detectable in the DRM fractions of this experiment.

Further analysis of the intracellular localization of K15 in stably infected HuARLT2-rKSHV cells by IFA staining using markers specific for different cytoplasmic compartments revealed the presence of K15 in vesicular structures in the perinuclear area. Here, K15 co-localized with a cis-Golgi network marker GM130 as well as a late endosome marker LAMP1 (Fig 4D). Consistent with the flotation assay experiment, only a portion of the K15 protein co-localized with the lipid raft marker Caveolin at the cell periphery while there was no clear localization to clathrin positive vesicles (Fig 4D).

**Fig 5:**
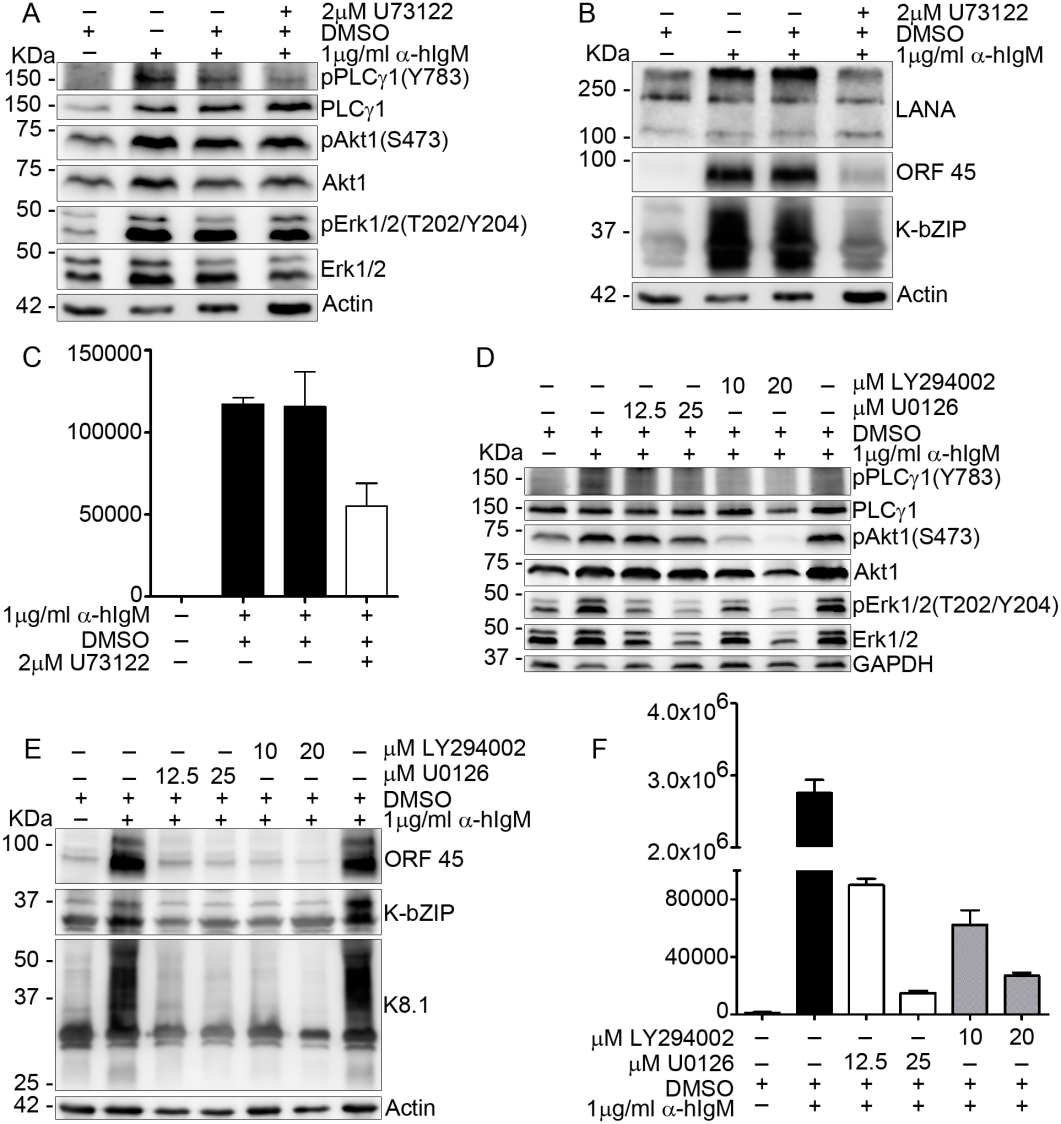
Inhibiting the activation of the PLCγ, MAPK and PI3K/Akt signaling pathways blocks KSHV reactivation. BJAB-rKSHV.219 cells were treated with the indicated inhibitors or DMSO as a control and the KSHV lytic cycle was induced with 1 μg/ml of anti-human IgM antibody applied together with the inhibitors. Seventy two hours after lytic induction, cells were lysed and the activation level of the indicated signaling components **(A and D)** as well as the expression level of lytic viral proteins **(B and E)** was assessed by western blot and **(C and F)** infectious virus titer was determined from the culture supernatant. Results are representative of three independent experiments. Bar graphs **(C and F)** represent the means ± SD of three replications.

In order to get further mechanistic insight into the role of K15-induced signaling in KSHV reactivation, BJAB-rKSHV.219 cells were treated with pharmacological inhibitors for the PLCγ (U73122), MAPK (U0126) and PI3K/Akt (Ly294002) pathways and the KSHV lytic cycle was induced by using anti-hIgM antibody. Treatment of these cells with 2 μM of U73122 decreased PLCγ1 phosphorylation in reactivated cultures; this was accompanied by reduced viral lytic gene expression and infectious virus release (Fig 5A–5C). Similarly, consistent with the literature (see Introduction), 12.5 μM of the MAPK inhibitor U0126 as well as 10 μM of the PI3K-Akt inhibitor Ly294002 efficiently inhibited KSHV lytic replication as shown both by the dramatic reduction in lytic gene expression as well as infectious virus production (Fig 5D–5F). In accordance with the results of the these experiments, depleting PLCγ1 from the stably infected HuARLT2-rKSHV cells by siRNA affected the ability of the virus to reactivate from latency as shown by the reduced number of RFP positive cells as well as lytic gene expression (S2 Fig). This observation confirms the involvement of PLCγ1 in KSHV reactivation in endothelial cells.

### Inhibiting the recruitment of PLCγ1 to K15 blocks KSHV reactivation

**Fig 6:**
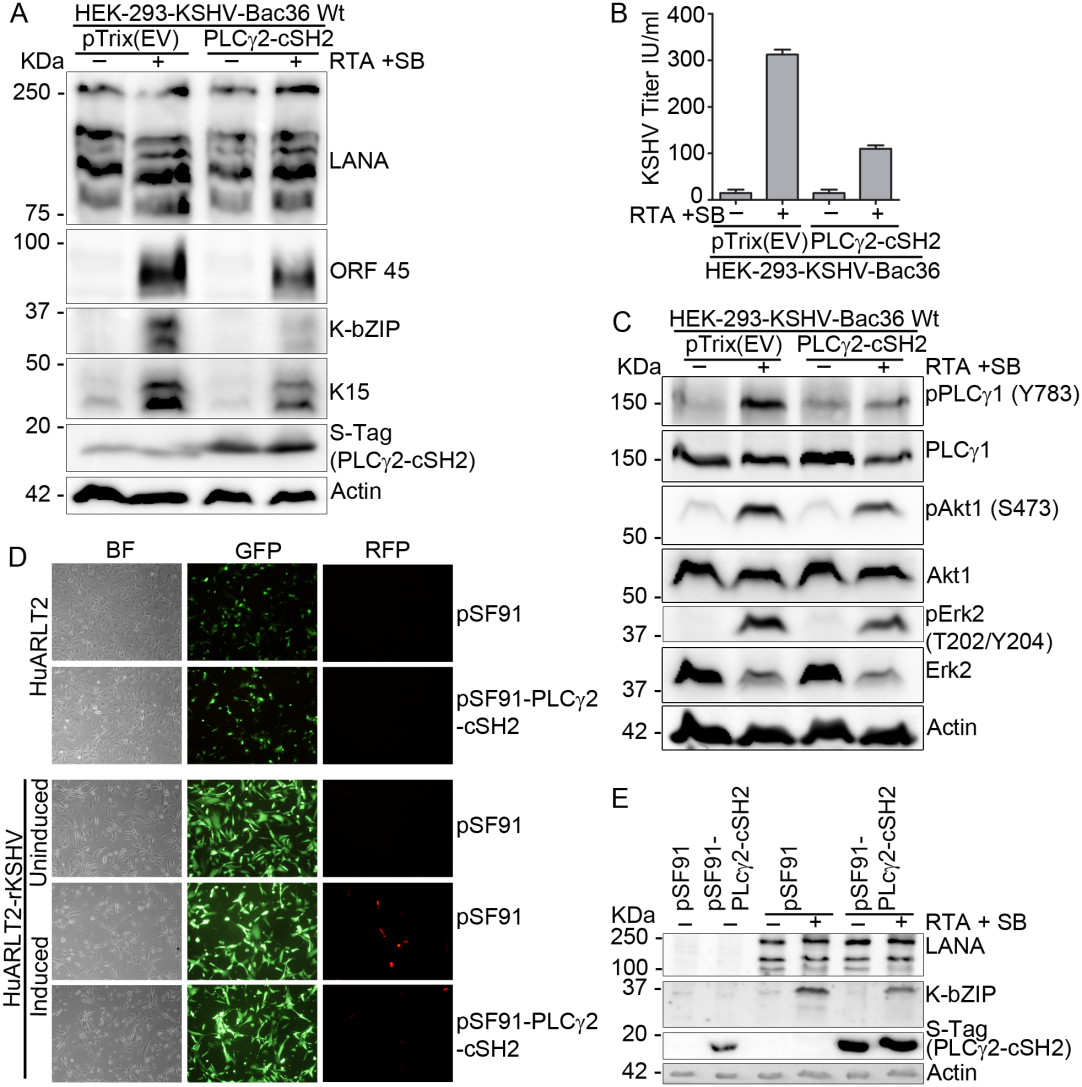
The isolated PLCγ2-cSH2 domain blocks KSHV reactivation. HEK-293-KSHV-Bac36 Wt cells were transfected with a plasmid expressing the PLCγ2-cSH2 domain or its empty vector control and the lytic cycle was induced 24 hours later. Cells and cell culture supernatant were collected 48 hours after induction of the lytic cycle; **(A)** expression level of KSHV lytic proteins, **(B)** virus titer in the supernatant and **(C)** phosphorylation level of the indicated signaling components was analyzed as described before. HuARLT2 or HuARLT2-rKSHV cells were transduced with a retrovirus vector expressing either the PLCγ2-cSH2 or the empty vector control and the KSHV lytic cycle was induced 24 hours later. Forty eight hours after induction of the lytic cycle, images were taken for GFP and RFP expression **(D)**, cells were lysed and expression level of the indicated viral proteins was analyzed by western blot **(E)**. Experiments were performed at least two times. Bar graphs in **(B)** represent the means ± SD of 2 replications.

Previously [49], we have shown that the isolated cSH2 domain of PLCγ2 can bind to the K15 protein and inhibit the recruitment of PLCγ1 to K15 in a dominant negative manner, thereby inhibiting K15-dependent PLCγ1 signaling, K15-induced angiogenesis and invasiveness. To investigate whether it could inhibit KSHV reactivation as well, a plasmid expressing the isolated PLCγ2-cSH2 domain or its empty vector control was transfected into the HEK-293 cells carrying the KSHV Bac36 wild type virus genome and its effect on KSHV reactivation was assessed. Expression of the PLCγ2-cSH2 domain in these cells inhibited virus reactivation as shown by the decreased expression level of the KSHV lytic proteins K-bZIP, ORF45 and K15 itself (Fig 6A), which was accompanied by reduced infectious virus release (Fig 6B). Transfection of the isolated PLCγ2-cSH2 domain also inhibited the activation of PLCγ1 (phosphorylation on Tyr783), Akt1 (Ser473) and Erk2 (Thr202/Tyr204) after induction of the KSHV lytic cycle compared to KSHV-carrying cells transfected with the empty vector (Fig 6C).

To extend this observation to endothelial cells, stably infected HuARLT2-rKSHV cells were transduced with a lentiviral vector expressing the isolated PLCγ2-cSH2 domain. Similar to the results in the epithelial cells, expression of the isolated PLCγ2-cSH2 domain abolished virus reactivation as shown by the reduced number of RFP (expressed under the control of the lytic PAN promoter) expressing cells (S3A Fig), and decreased K-bZIP and ORF 45 expression (S3B Fig), as well as reduced infectious virus release (S3C Fig) compared to cells transduced with an empty vector control. The isolated PLCγ2-cSH2 domain was also cloned into a retroviral vector (pSF91-IRES-GFP) which, unlike the lentivirus vector, can express GFP to allow the monitoring of transduction efficiency. The stably infected HuAR2T-rKSHV cells were then transduced with this retroviral vector expressing the PLCγ2-cSH2 (pSF91-PLCγ2-cSH2-IRES-GFP) or with the empty vector as a control. Expression of the isolated PLCγ2-cSH2 domain from the retroviral vector also inhibited KSHV reactivation as shown by the reduced RFP (Fig 6D lower panel) and K-bZIP expression (Fig 6E) compared to cells transduced with the empty vector; equal transduction efficiency of both retroviral stocks was assessed based on GFP expression in uninfected HuAR2T cells that had been transduced with the pSF91-PLCγ2-cSH2-IRES-GFP or control retroviral vector in parallel (Fig 6D upper panel).

### K15 is required for KSHV lytic replication in stably infected LECs and plays an important role in virus induced endothelial-spindle cell formation

**Fig 7:**
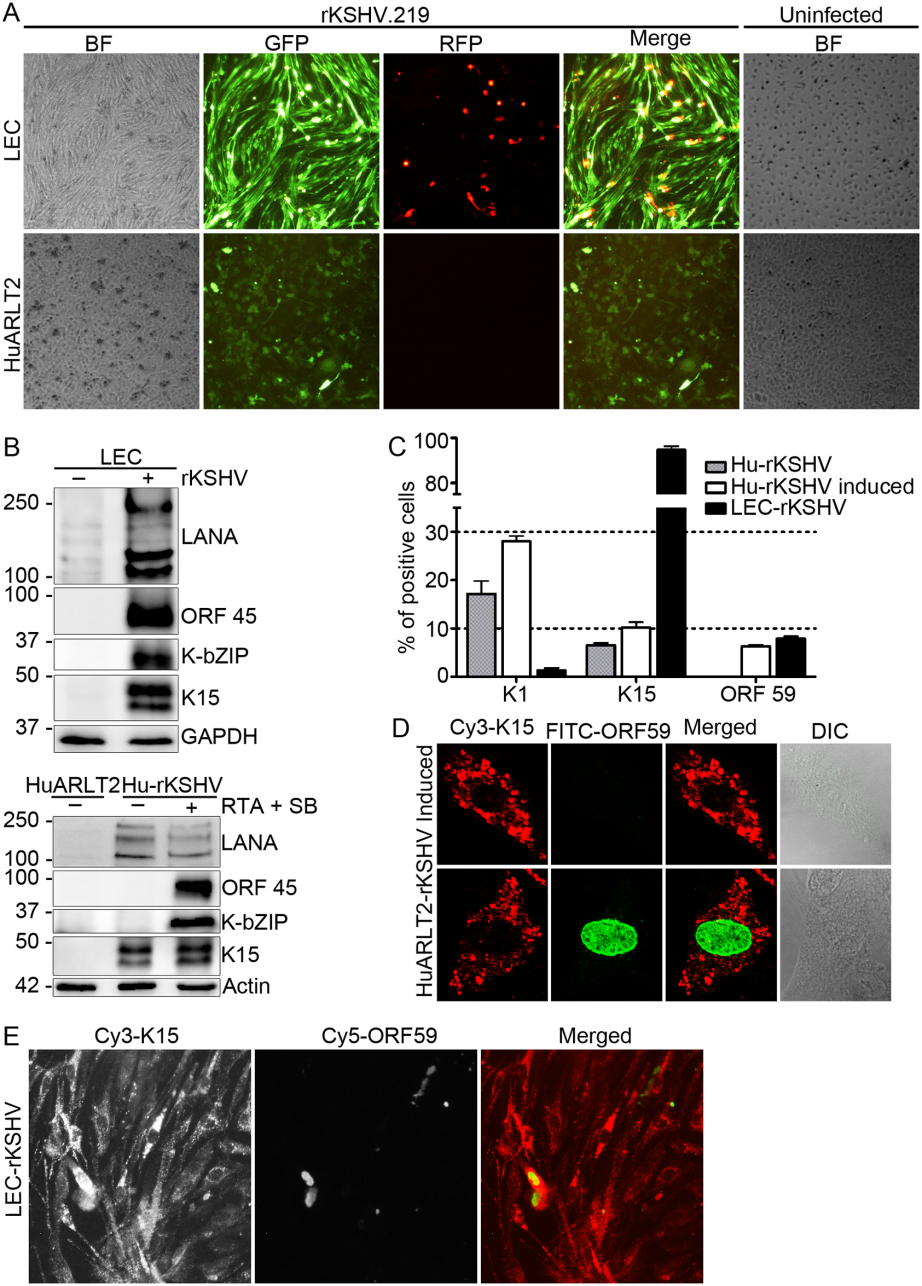
K15 protein expression in KSHV stably infected LEC and HuARLT2 cells. LEC and HuARLT2 cells were infected with rKSHV.219 virus at MOI 1 and **(A)** images were taken 7 days after infection. Two weeks post infection, cells were collected and **(B)** the expression level of the indicated viral lytic proteins in the stably infected LECs (upper panel) or HuARLT2 cells (lower panel) before and after lytic induction was analyzed by western blot. **(C)** HuARLT2-rKSHV cells with or without induction of the KSHV lytic cycle or stably infected LEC-rKSHV cells were stained with a mouse anti-K1 mAb, a rat anti-K15 mAb or a mouse anti-ORF59 mAb followed by a Cy3-conjugated anti-mouse or anti-rat IgG; the number of cells expressing K1, K15 or ORF 59 protein was manually counted. Bar graphs represent the means ± SD of 3 replications. **(D)** HuARLT2-rKSHV cells after lytic reactivation were co-stained with a rat anti-K15 mAb and a mouse anti-ORF 59 mAb followed by a Cy3-conjugated anti-rat (red) and FITC-conjugated anti-mouse (green) secondary antibodies and the representative images were acquired using a Leica confocal microscope. **(E)** LEC-rKSHV cells were co-stained with a rat anti-K15 mAb and a mouse anti-ORF 59 mAb followed by a Cy3-conjugated anti-rat (red) and Cy5-conjugated anti-mouse (green) secondary antibodies. Images for **(C)** and **(E)** were acquired using a Zeiss Observer.Z1 epi-fluorescent microscope.

Chang and Ganem have recently described a unique KSHV transcription program with a widespread expression of both latent and lytic genes in stably infected lymphatic endothelial cells (LECs), which differs from the traditional latency program observed in stably infected blood endothelial cells such as HUVECs [68]. The fact that these cells support lytic replication without the need for ectopic RTA expression or treatment with chemical inducers such as sodium butyrate may permit the investigation of the KSHV lytic cycle in LECs in a perhaps more physiological model. To investigate the role of K15 in this regard, LECs as well as HuARLT2, an endothelial cell line derived by immortalizing HUVECs, were infected with the recombinant rKSHV.219 virus and maintained under puromycin selection for two weeks to generate stably infected polyclonal cell populations, LEC-rKSHV and HuARLT2-rKSHV. KSHV infection of endothelial cells in culture is known to induce a change from their typical coble-stone morphology into an elongated spindle shape, a phenotypic change known as spindling [69], which is similar to the appearance of KS spindle cells in KS lesions. Microscopic examination of the infected cells revealed that KSHV induced extensive spindling in LEC cells, which was already visible after 48 hours of infection (see images taken 7 days after infection shown in Fig 7A), while HuAR2T-rKSHV cells did not show an obvious change in their morphology; the level of infection is shown by the expression of GFP in both cell populations (Fig 7A). Additionally, the LEC-rKSHV cells are also expressing the lytic marker RFP a week after infection indicating that there is still ongoing lytic replication (Fig 7A upper panel). In contrast, lytic gene expression in the HuARLT2-rKSHV cells has subsided one week after infection (Fig 7A lower panel), but can be induced using RTA and SB (S4 Fig). Consistent with the RFP expression, the KSHV lytic proteins K-bZIP and ORF 45 are expressed in the LEC-rKSHV cells after two weeks of infection as shown by western blot analysis (Fig 7B upper panel); in contrast, in HuARLT2-rKSHV cells, this can only be seen after lytic reactivation with RTA and SB (Fig 7B lower panel). These findings are in line with those reported by Chang and Ganem [68]. Analysis of the expression of the K15 protein in the stably infected HuARLT2-rKSHV cells (Fig 7B lower panel) revealed that, unlike K-bZIP and ORF 45, which are expressed only after lytic induction, K15 protein is already detectable in latently infected cells and its expression increases upon lytic reactivation in a similar fashion to what was observed in HuARLT2 cells infected with KSHV Bac36 virus (see Fig 2E). Interestingly, a similar experiment in the stably infected LEC-rKSHV cells revealed an abundant K15 expression (Fig 7B upper panel).

Further, we performed immunofluorescence (IF) staining of endogenous K1 and K15 proteins as well as ORF 59 (KSHV DNA polymerase processivity factor-8/PF-8) as a lytic marker in the stably infected LEC-rKSHV or HuARLT2-rKSHV cells with or without induction of the lytic cycle. The result (Fig 7C) showed that a small fraction of HuARLT2-rKSHV cells expresses the K1 or K15 proteins, 17% and 6.5% respectively, which increased to 28% and 10% respectively upon induction of the lytic cycle. In contrast, the majority of the LEC-rKSHV cells were expressing the K15 protein while the number of K1 expressing cells is negligible (Fig 7C) in agreement with the absence of detectable K1 mRNA expression in the previously reported KSHV tiling microarray study on KSHV-LECs [68]. Unlike the K1 and K15 proteins, ORF 59 is expressed only after induction of the lytic cycle in 6% of HuARLT2-rKSHV cells, while it is already present in 8% of LEC-rKSHV cells without any stimulation for lytic reactivation (Fig 7C).

Next, we co-stained the K15 and ORF 59 proteins by IFA in HuARLT2-rKSHV cells, in which the KSHV lytic cycle was induced using KSHV RTA and SB. Interestingly, ORF 59 staining was evident only in the K15 expressing cells, while the reverse is not true (representative IF images of cells expressing K15 alone or together with ORF 59 are shown in Fig 7D upper and lower panel respectively), in agreement with the greater number of cells (10%) expressing the K15 protein compared to the number of cells expressing ORF 59 (6%) during lytic replication (Fig 7C). Moreover, K15 expression in lytic (ORF 59 co-expressing) cells shows a dispersed and peripheral localization (Fig 7D) from its perinuclear abundance in vesicular structures in the non-lytic cells (Figs 7D and 4D). Similar to the result in the induced HuARLT2-rKSHV cells, no detectable ORF 59 expression was observed in the absence of K15 expression in the stably infected LEC-rKSHV cells (representative IF images are shown in Fig 7E), indicating that K15 expression in the infected cell might be a pre-requisite for KSHV lytic replication.

**Fig 8:**
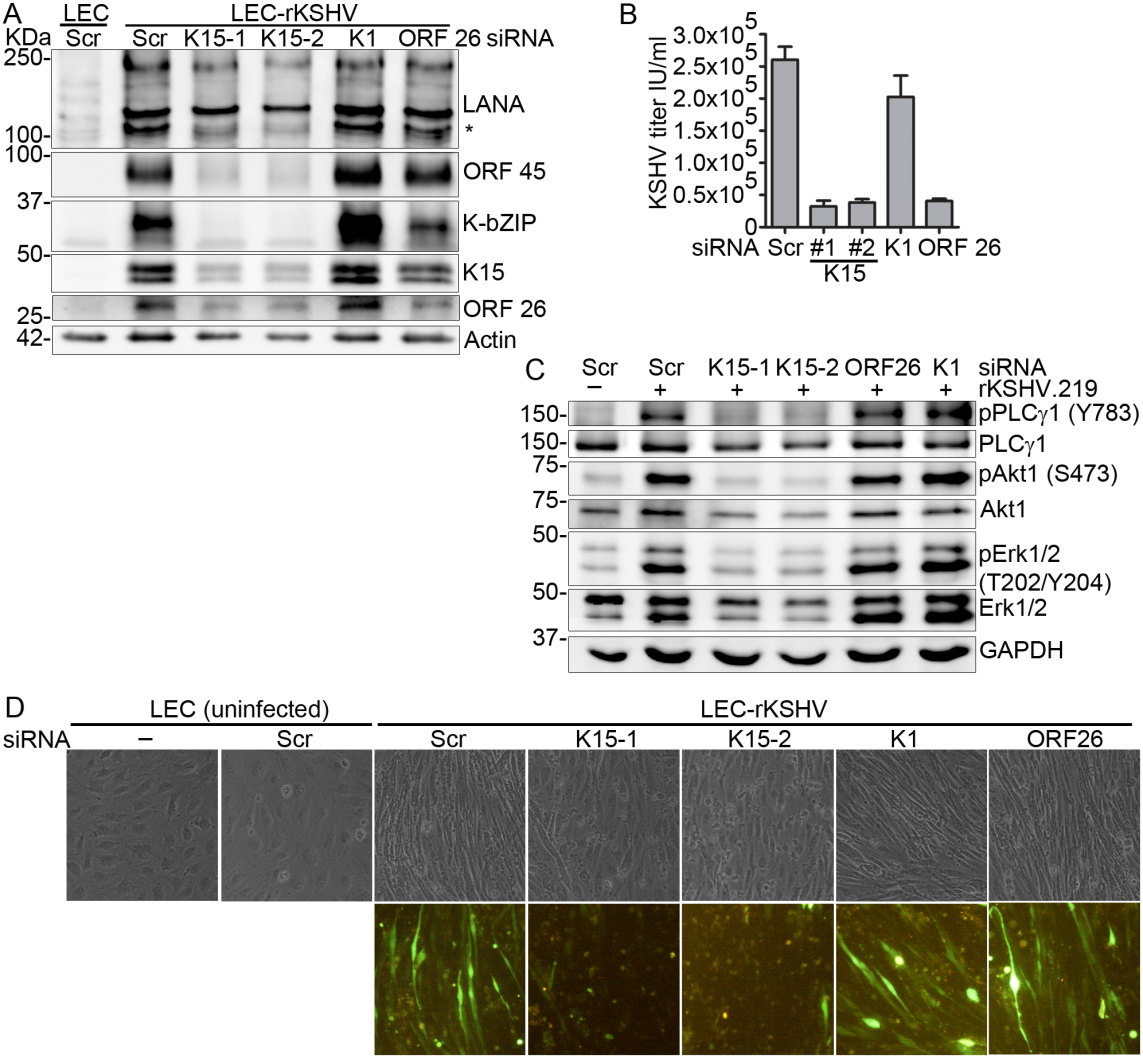
Depletion of K15 from stably infected LECs impairs KSHV lytic replication and reverses endothelial cell spindling. LEC or LEC-rKSHV cells were transfected with the indicated siRNAs. Seventy-two hours after siRNA mediated knock-down, cells and cell culture supernatant were collected; **(A)** western blot of KSHV lytic proteins, **(B)** KSHV infectious virus titer, **(C)** western blot analysis of signaling components and **(D)** representative images of siRNA transfected cells taken before cell lysis. Results are representative of two or more independent experiments. Bar graphs in **(B)** represent the means ± SD of 3 replications. * Low molecular weight isoforms of LANA (see text).

We then asked whether the abundant expression of K15 in the stably infected LEC-rKSHV cells could contribute to the lytic gene expression program and ongoing lytic replication in these cells. To investigate this question, stably infected LEC-rKSHV cells were transfected with either a scrambled (Scr) control siRNA or two siRNAs against K15 or a single siRNA against K1; siRNA targeting the mRNA for the small capsid protein encoded by ORF 26 was included as a control for a late lytic gene which affects only virus production but not early lytic viral gene expression. First, we investigated the effect of K15 depletion on KSHV lytic replication by western blot analysis of the expression of K-bZIP and ORF 45 as well as LANA proteins. The result (Fig 8A) shows that silencing the K15 mRNA in LEC-rKSHV cells results in a pronounced decrease of K-bZIP and ORF 45 expression as well as the low molecular weight isoform of LANA, while the higher molecular weight isoforms of LANA were unaffected. Transfection of the siRNA against K1 did not reduce lytic gene expression in KSHV-infected LECs (Fig 8A); this is in line with the absence or low expression of K1 in these cells (see Fig 7C). Likewise, as expected, silencing ORF 26 mRNA did not affect early lytic gene expression (Fig 8A). As reported previously [68], we also found that KSHV-infected lymphatic endothelial cells can, in addition to showing an extended lytic gene expression program, also release substantial titers of infectious KSHV (Fig 8B). Silencing K15 mRNA in these cells abrogated infectious virus release to the level of knocking down the capsid protein ORF 26, while transfection with siRNA against K1 did not affect virus production (Fig 8B). Further, western blot analysis of the phosphorylation level of PLCγ1, Akt1 and Erk1/2 in the LEC-rKSHV cells revealed that all three pathways are activated in the stably infected cells compared to the uninfected control cells (Fig 8C, lanes 1 and 2). Phosphorylation at the indicated residues of PLCγ1, Akt1 and Erk1/2 was reduced in response to K15 silencing but not in cells transfected with siRNAs against either K1 or ORF 26 (Fig 8C).

**Fig 9:**
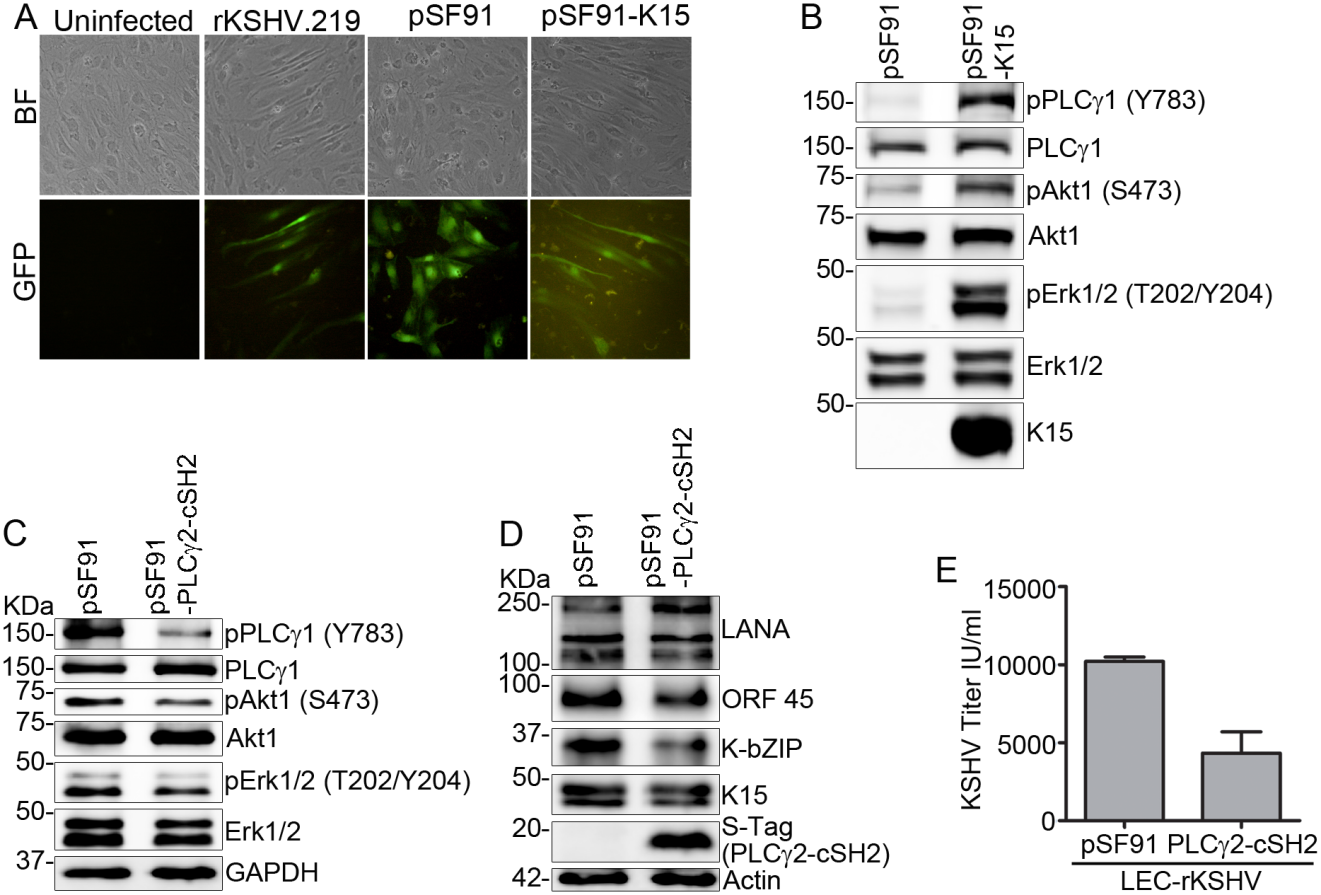
K15 expression in LECs induces signaling activation and endothelial cell spindling similar to KSHV infection. LECs were transduced with a retrovirus vector expressing K15 or an empty vector control, or in parallel infected with rKSHV.219 virus or left uninfected. After 48 hours **(A)** representative images were taken and **(B)** the activation level of the indicated signaling components was assessed by western blot. **(C)**—**(E)** stably infected LEC-rKSHV cells were transduced with a retrovirus vector expressing the PLCγ2-cSH2 or an empty vector control. Seventy two hours later, cells and cell culture supernatant were collected; **(C)** activation of signaling components and **(D)** expression level of lytic viral proteins were analyzed by western blot, and **(E)** KSHV infectious titer was analyzed as described before. Bar graphs in **(E)** represent the means ± SD of 2 replications.

Interestingly, in addition to inhibiting the lytic transcription in the stably infected LEC-rKSHV cells, K15 depletion also led to the loss of the pronounced spindle cell morphology induced by KSHV infection (Fig 8D); in contrast, we did not observe an obvious phenotypic change after transfecting siRNAs against either K1 or ORF 26 (Fig 8D). In support of a role for K15 in spindle cell formation, we also show that ectopic expression of K15 alone in LECs can induce morphological change similar to KSHV infection (Fig 9A) and this is accompanied by the activation of PLCγ1, Akt1 and Erk1/2 signaling pathways (Fig 9B). This suggests a direct role of K15 in the activation of these pathways in the stably infected LEC-rKSHV cells in agreement with the reduced phosphorylation of PLCγ1, Akt1 and Erk1/2 following K15 silencing in KSHV-infected LECs (Fig 8C). We could also show that inhibiting the K15-PLCγ1 interaction by transducing the retroviral vector for the PLCγ2-cSH2 domain (see above) as a dominant negative inhibitor can reduce the phosphorylation of PLCγ1, and, to a lower extent, Akt1 and Erk1/2 (Fig 9C), viral lytic protein expression (Fig 9D) and infectious virus release (Fig 9E) in the stably infected LEC-rKSHV cells, consistent with our results obtained with the HEK-293-Bac36 and HuARLT2-rKSHV cells (see above).

### K15 protein is abundantly expressed in KS biopsies from HIV positive patients

**Fig 10:**
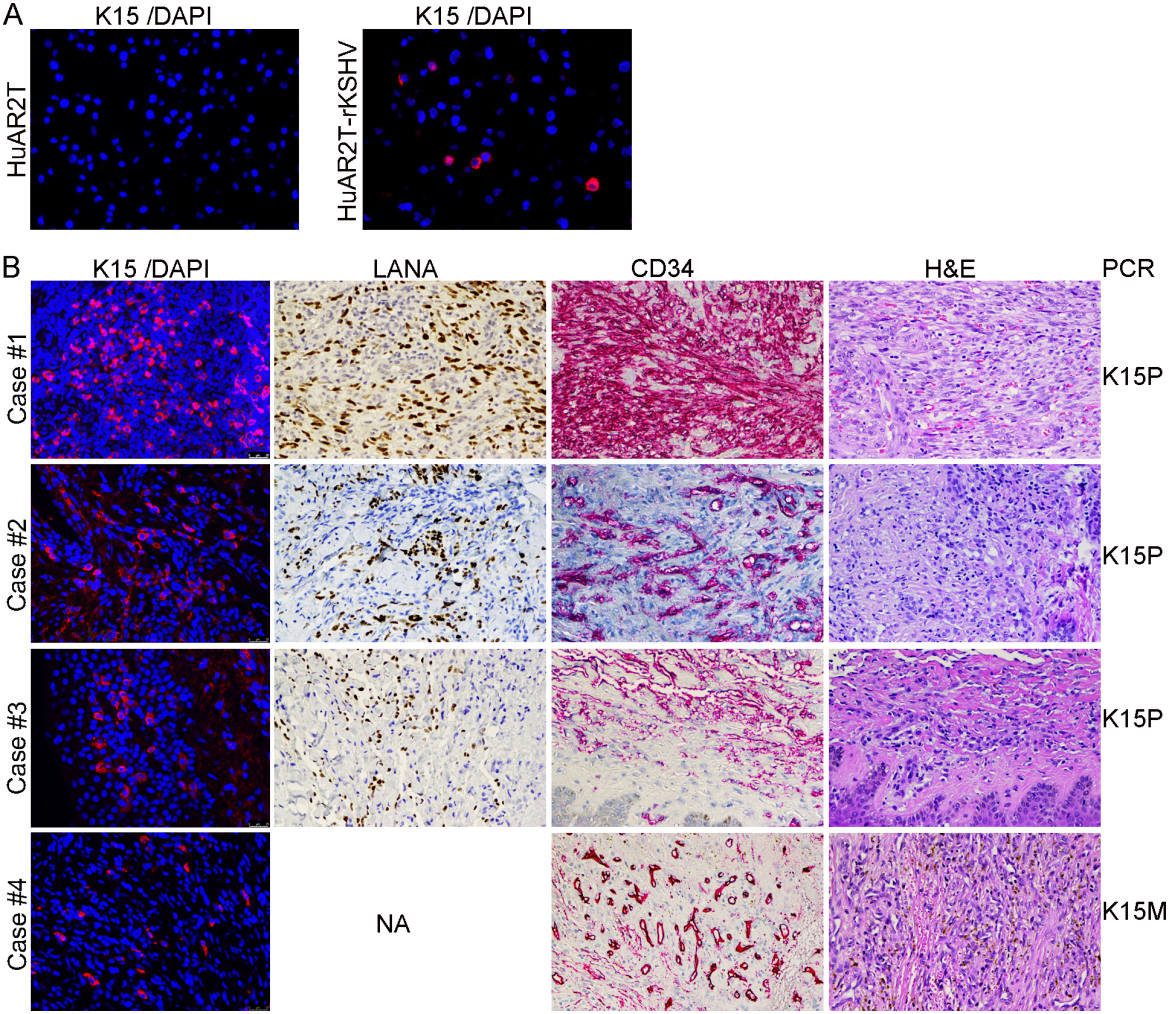
K15 is abundantly expressed in KS tissue biopsies. **(A)** IF staining, using antibody clone number 18E5, of K15 (red) in sections prepared from paraffin embedded blocks of HuARLT2 or HuARLT2-rKSHV cells. **(B)** KS tissue biopsies obtained from HIV positive individuals were stained for K15/DAPI by IF (first column), LANA (dark brown nuclei), CD34 (red) and Hematoxylin and Eosin (last column); the respective KSHV genotype (K15P or M) was determined by PCR and is shown on the right. NA—staining not available because of insufficient biopsy material.

Hosseinipour and colleagues have recently studied the transcription profile of KSHV in KS tissue biopsies from treatment-naïve HIV-positive patients by using a KSHV real-time quantitative PCR array with multiple primers per open reading frame [70]. In this study, the K15 mRNA was shown to be expressed as abundantly as the genes in the viral latency locus (LANA, vCyc, vFLIP, Kaposin, and miRNAs) in KS tissue biopsies with both a restricted and relaxed KSHV transcription profile [70]. However, K15 protein expression depends on post-transcriptional regulation exerted by KSHV ORF 57 [71]. Owing to the lack of suitable antibodies, it has not been possible so far to investigate whether the K15 protein is expressed in KS lesions. Here, the suitability of our rat anti-K15 monoclonal antibody (clone 18E5) [49] for staining paraffin-embedded tissue blocks was first tested on paraffin embedded cell blocks prepared from HuARLT2 or HuARLT2-rKSHV cells representing K15 negative and positive samples respectively. As shown in Fig 10A, when used with a tyramide signal amplification protocol, this antibody detects a cytoplasmic K15 staining in a fraction of the cells in the HuARLT2-rKSHV cell block which is consistent with its expression profile in our IFA experiments. In contrast, there is no detectable signal in the HuARLT2 cell blocks indicating the specificity of our staining protocol. Next, we applied this staining protocol to 9 KS tissue biopsies obtained from HIV positive patients. In this experiment, we also stained for KSHV LANA as a marker of infection, the endothelial cell marker CD34 as well as Hematoxylin and Eosin (H&E) to visualize tissue structures. Fig 10B shows four examples in which the K15 protein is expressed at a varying level of abundance, mirroring the abundance of LANA positive cells, in these KS tissues. LANA immunostaining for the fourth case is not shown because of the small size of the paraffin block in this case. We also observed a similar K15 expression of varying abundance in the remaining 5 analyzed tissue samples; however, because of the small size and low quality of these tissue biopsies, we could not stain for LANA as well as CD34 and H& E and therefore did not include them in Fig 10B. These findings are consistent with the K15 mRNA expression profile in KS biopsies described recently [70].

Genotyping of the KSHV genome extracted from these KS tissue biopsies using primers specific for the K15P and M alleles revealed that 7 out of the 9 samples were carrying the K15P type allele while 2 samples contained the less common K15M allele (Fig 10B). Interestingly, we were also able to detect the K15M protein using our monoclonal antibody in a KS biopsy that was positive for the K15M allele (Fig 10B last panel). Despite the fact that the two K15 protein forms are very divergent in their amino acid sequence, they contain conserved sequence motifs (see Introduction). Therefore, we analyzed the K15 epitope recognized by the monoclonal antibodies clone 10A6 (used in western blot) and 18E5 (used in IFA and IHC staining) by using a synthetic peptide array of 44 peptides derived from the K15 cytoplasmic tail sequence and overlapping with 3 amino acids. The last two peptides (number 43 and 44) with the peptide sequence ATQPTDDLYEEVLFP and PTDDLYEEVLFPRN respectively reacted with both monoclonal antibodies (S5A and S5B Fig). Although it was raised against a recombinant GST-tagged cytoplasmic domain of the K15P type protein, our monoclonal antibody recognizes one of these conserved motifs (the protein sequence PTDDLYEEVLFP), including and flanking the SH2 domain-binding site YEEVL at the c-terminal end of the K15 cytoplasmic tail (S5A and S5B Fig). To confirm that this antibody could also detect proteins encoded by the K15M type allele, we transfected a plasmid expressing either of the two K15 forms and performed IFA. Indeed, both K15P and M proteins were recognized by our monoclonal antibody clone 18E5 (S5C Fig).

## Discussion

The majority of KS endothelial spindle cell in KS tumors is latently infected with KSHV and there is substantial evidence that several latent KSHV genes, i.e. LANA [72, 73], vCyc [74, 75], vFLIP [76, 77] and the KSHV miRNAs [78, 79] may contribute to the increased survival and proliferation of infected endothelial cells. In addition, several early KSHV genes, such as vIL6 and vGPCR, as well as productive replication and seeding of KSHV into new cells are important for the pathogenesis of KS [7, 80–83]. In keeping with this, a small fraction of KS spindle cells in advanced KS lesions express lytic viral proteins [6, 8, 84–86], most KS lesions display a viral gene expression pattern that includes early lytic genes [70] and release of angiogenic and inflammatory factors from such type of cells during lytic replication have been suggested to create a conducive microenvironment for the development of KS [5, 9, 82, 83].

In this study, we investigated the role of the K15 protein in the virus life cycle and in KSHV-induced pathogenesis. Having previously shown that K15 contributes to the increased invasiveness and angiogenic properties typical for KSHV-infected endothelial cells by recruiting and activating PLCγ1 [48, 49], we now provide evidence that K15 uses the same signaling pathway to support the activation of the early stages of the lytic cycle. We also provide proof of principle that the recruitment of PLCγ1 by K15 may represent a novel therapeutic target to silence KSHV reactivation (Figs 6 and 9). Furthermore, we demonstrate the expression of the K15 protein in a substantial proportion of KSHV-infected endothelial spindle cells in KS lesions (Fig 10) as a prerequisite for defining K15 as a novel therapeutic target. Both by siRNA-mediated silencing as well as deleting the K15 gene from the viral genome we show, in a variety of KSHV infected cells, that the expression of K15 is crucial for reactivation from latency, early viral gene expression and virus production (Figs 1, 2 and 8). K15 expression is required earlier during lytic replication as demonstrated by the reduced expression of immediate early lytic viral proteins including the master lytic switch protein RTA, ORF 45 and K-bZIP upon K15 knock down in stably infected cells or when K15 is deleted from the viral genome (Figs 1A, 2B and 2E). The decreased expression of these immediate-early/ early viral proteins RTA, ORF 45 and K-bZIP in the absence of K15 can further affect the expression of late lytic proteins, such as the envelope glycoprotein K8.1 (Fig 1A) and therefore lead to reduced virus production (Figs 1B, 2C and 2F).

We also compared the role of K15 to that of K1, which has previously been shown to be required for efficient viral reactivation [59, 87, 88]. Interestingly, while confirming these earlier reports for epithelial and blood vascular endothelial cells (Fig 2), we also found that K1 is only expressed at low levels, and may therefore not contribute to KSHV-reactivation, in infected lymphatic endothelial cells (Figs 7 and 8). KSHV infected lymphatic endothelial cells are spontaneously lytic [68] and their cellular transcriptome profile has been shown to resemble that of KS biopsies [89, 90]. They may therefore reflect the *in vivo* situation in KS tumors better than KSHV-infected blood vascular endothelial cells, in which KSHV tends to be more latent (Fig 7). In keeping with this notion, the previously reported expression of K15 mRNA in many KS biopsies from AIDS patients [70], as well as the presence of activating epigenetic marks on the K15 genomic locus [91], we show here that K15 is spontaneously expressed in KSHV-infected blood vascular (HuARLT2) and lymphatic endothelial cells (Figs 2E and 7–9), as well as in a substantial proportion of KS spindle cells in tumor biopsies (Fig 10). In cultured endothelial cells, the number of K15 positive cells vastly exceeds that of cells undergoing lytic viral DNA replication, as indicated by expression of the viral polymerase-associated factor encoded by ORF 59; in addition, ORF 59-positive cells are always positive for K15, while the reverse is not the case (Fig 7). It appears therefore possible that K15 expression ‘primes’ endothelial cells for the onset of lytic replication but is not sufficient for this process to initiate.

In addition to K1 and K15, the viral G-protein coupled receptor (vGPCR) has been shown to positively regulate viral lytic replication [92, 93]. All three viral membrane proteins exert their roles by activating intracellular signaling pathways, in particular PLCγ, Erk1/2, Akt1 or NF-κB [64, 87, 88, 92, 93]. We show here that the absence of K15 impedes the activation of PLCγ1, Erk1/2 and Akt1 (Figs 3C, 3D and 8C), and that these pathways are also directly activated by K15, at least in the lymphatic endothelial cells (Figs 3B and 9B). We believe that in HEK-293 cells, in which Akt is not activated by K15 (Fig 3B), the decreased expression of other viral proteins such as K1 and vGPCR account for the lack of Akt1 activation in the absence of K15 (Fig 3C and 3D). In fact, our results suggest that deleting K15 is accompanied by reduced K1 expression upon viral lytic reactivation (Fig 2).

The phenotypic similarity of the K1 and K15 deletion mutant viruses in regard to virus reactivation as well as intracellular signaling activation (Figs 2 and 3) led us to investigate their intracellular localization. In the context of viral infection, we found both viral membrane proteins to be predominantly localized in intracellular vesicular structures rather than at the cell surface membrane (Fig 4A and 4D). Overexpressed K15 is known to localize to DRMs (lipid rafts) [45]. We show here that, in KSHV infected cells, a portion of K15 is also associated with lipid rafts at the cell periphery while a substantial amount localizes into the perinuclear area in GM130 and LAMP1 positive vesicles (Fig 4B–4D).

Not only did K15 depletion affect viral replication in KSHV-infected lymphatic endothelial cells, it also reversed the virus-induced endothelial cell spindling (Fig 8D), and K15 overexpression in these cells recapitulates KSHV infection with regard to spindle cell formation (Fig 9A). Previous work [94, 95] has shown the latent KSHV protein viral FLIP (vFLIP) to be crucial in this regard through its activation of the NF-κB pathway [68]. K15 can also mediate NF-κB activation [45, 96, 97], suggesting that K15 induced LEC spindling may also be the result of NF-κB activation. Activation of the PLCγ1 and MAPK pathways (Fig 9B) which can also affect the expression of genes involved in cytoskeleton reorganization could also contribute to this phenotype.

In view of our observation (Figs 7 and 8) that, the absence of K15 also impedes the spontaneous lytic reactivation observed in KSHV-infected lymphatic endothelial cells, we explored if K15 might serve as a potential therapeutic target to interfere with KSHV early gene expression and lytic replication. We found that overexpression of the PLCγ2-cSH2 domain, which we have previously shown to interfere with the recruitment of PLCγ1 to K15 [49] can inhibit PLCγ1, Erk1/2 and Akt1 phosphorylation, the expression of early viral proteins and virus production in KSHV-infected epithelial and endothelial cells (Figs 6 and S3), as well as in the spontaneously lytic lymphatic endothelial cells (Fig 9). These results suggest that, if a small molecule inhibitor of the K15-PLCγ1 interaction could be developed, it might be used to interfere with KSHV early gene expression and thereby its role in the pathogenesis of Kaposi’s Sarcoma.

## Materials and methods

### Ethics statement

Written informed consent obtained from all patients covered the use of human biopsies, taken for diagnostic purposes, for this study and was approved by the Hannover Medical School Ethics Committee (Approval Nr. 3381–2016). Experiments were conducted in accordance with the Declaration of Helsinki.

### Antibodies

The list of primary and secondary antibodies used for western blot analysis is shown below. The rabbit anti-KSHV RTA polyclonal antibody [98] was a kind gift from David Lukac (Rutgers new Jersey Medical School, Newark, New Jersey, USA). The production of the rat anti-KSHV K15 monoclonal antibody (clone number 10A6) was described before [49] and the mouse anti-KSHV K1 monoclonal antibody (Clone 3H4) [65] was kindly provided by Jae U. Jung (University of Southern California, Los Angeles, California, USA). Primary antibodies rabbit anti-GAPDH (#14C10), rabbit anti-S-Tag (#8476), rabbit anti-PLCγ1 (#2822), rabbit anti-phospho PLCγ1 (Tyr783; #2821), mouse anti-phospho MAP-Kinase p44/42 (Thr202/Tyr204; #91062), rabbit anti-phospho Akt1 (Ser473; #4058), mouse anti-Akt1 (#2967) and mouse anti-MAP-Kinas p44/42 (#9107) were purchased from Cell Signaling Technology; mouse anti-Erk2 (D-2) (sc-1647), mouse anti-KSHV ORF 45 (sc-53883) and mouse anti-KSHV K-bZIP (sc-69797), were purchased from Santa Cruz Biotechnolocy Inc.; mouse anti KSHV K8.1 (A2B) (13-212-100), rat anti-KSHV ORF73 (LNA-1;13-210-100) and mouse anti-KSHV ORF 59 (13-211-100) were purchased from Advanced Biotechnology Inc.; mouse anti-HHV-8 ORF26 (LS-C41403) was purchased from LifeSpan Biosciences Inc.; mouse anti-β-actin (A5441) was purchased from Sigma Aldrich; and rabbit anti-caveolin (610060) was purchased from BD Transduction Laboratories. HRP-conjugated secondary antibodies: goat anti-rabbit IgG (P0448), rabbit anti-mouse IgG (P0260) and goat anti-mouse IgG (P0447) were purchased from DAKO; goat anti-rat IgG (#3050–05) was purchased from SouthernBiotech.

The following primary and secondary antibodies were used for IFA. The production of the rat anti-KSHV K15 monoclonal antibody (clone 18E5) was described before [49]. Here, the K15 epitope necessary for antibody binding for both clones used in western blot (clone 10A6) and IFA (clone 18E5) was mapped by using a microarray of 44 overlapping synthetic peptides from the K15 cytoplasmic tail sequence with 3 amino acid shifts as described before [99]. In brief, the peptides were synthesized using the SPOT method on a soluble cellulose matrix [100]. After dissolving, the cellulose-peptide conjugates were printed to glass slides via the SC^2^ process [101], resulting in 8 arrays per slide. The microarrays were blocked with 2% (w/v) casein in TBS-T overnight at room temperature, followed by incubation with primary antibodies (10A6 and 18E5) in a dilution of 1:100 in blocking buffer overnight at 4**°C.** After three washes with TBS-T, staining of bound antibodies and control spots was carried out at room temperature via incubation with secondary antibodies (Cy3-anti-rat IgG conjugate and Streptavidin-Cy5) at a dilution of 1:240 for 90 min. The slides were then washed three times with TBST and two times with dH_2_O for 5min each, dried in a stream of nitrogen and scanned with an Agilent microarray scanner (Agilent Technologies, Inc., Santa Clara, CA, USA).

The mouse anti-KSHV K1 monoclonal antibody (Clone 2H5) [65] was kindly provided by Jae U. Jung (University of Southern California, Los Angeles, California, USA); and the mouse anti-KSHV ORF 59 antibody (13-211-100) was purchased from Advanced Biotechnology Inc. The following fluorescently labelled secondary antibodies were used: FITC-conjugated donkey anti-rabbit IgG (711-095-152; Jackson Immuno Reasearch), Cy3-conjugated donkey anti-rat IgG (712-165-153; Jackson Immuno Reasearch), Cy3-conjugated donkey anti-mouse IgG (715-165-151; Jackson Immuno Reasearch), Streptavidin-Cy5 (016-170-084; Jackson Immuno Research), and Cy5-conjugated goat anti-mouse IgG (115-175-164; Jackson Immuno Research).

### Cells, viruses, infection and transfection

HEK-293 (ATCC, CRL-1573), HEK-293T (DSMZ No.: ACC 305) and HeLa CNX (DSMZ No.: ACC 57) cells were maintained in Dulbecco’s modified Eagle medium (DMEM; Gibco, lifetechnologies, Paisley, UK) supplemented with 10% heat inactivated fetal bovine serum (FBS; Hyclone, Cramlington, UK). BJAB-rKSHV.219 cells are BJAB cells (DSMZ No.: ACC 757) stably infected with rKSHV.219, a recombinant virus derived from JSC-1 cells that expresses the red fluorescent protein (RFP) from the KSHV lytic gene PAN promoter, the green fluorescent protein (GFP) from the cellular EF-1α promoter, and a puromycin resistance gene as a selectable marker [61] and have been described previously [53]; they were grown and maintained in RPMI 1640 medium (Gibco, lifetechnologies, Paisley, UK) supplemented with 20% heat inactivated FBS in the presence of 4.2 μg/ml puromycin (A2856; Aplichem GmbH, Darmstadt, Germany). Primary juvenile foreskin lymphatic endothelial cells (LECs; Lonza) were provided by Päivi M. Öjala (University of Helsinki, Helsinki, Finland) and grown in EGM-2MV medium (Lonza, Walkersville, MD, USA). HuARLT2, a conditionally immortalized human endothelial cell line derived from HUVECs by expressing doxycycline-inducible transgenes simian virus 40 (SV40) large T antigen (TAg) and human telomerase reverse transcriptase (hTert) [62], was kindly provided by Dagmar Wirth (HZI, Braunschweig, Germany) and was grown and maintained in EGM-2MV medium in the presence of 1 μg/ml doxycycline (D9891; Sigma-Aldrich, Saint Luis, Missouri, USA). HuARLT2-rKSHV.219, HuARLT2 cell line stably infected with rKSHV.219 as described elsewhere in [94], was grown and maintained in EGM-2MV medium in the presence of 1 μg/ml doxycycline and 5 μg/ml puromycin.

rKSHV.219 virus was produced from BJAB-rKSHV.219 cells as described before [49, 53]. Briefly, 6 X 10^5^ BJAB-rKSHV.219 cells/ml were inoculated and grown for four to five days in TECHNE spinner flasks (Cole-Parmer GmbH, Wertheim, Germany) with 60 rpm agitation in the presence of 2.5 μg/ml of anti-human IgM antibody (#2020–01; SoutherBiotech) to induce the KSHV lytic cycle. Culture supernatant was collected after low speed centrifugation to remove cells and debris, filtered through a 0.45 μm filter and virus particles were concentrated by ultracentrifugation at 15,000 rpm for 5 hours in a Type 19 rotor (Beckman Coulter Inc., Brea, CA, USA). The pellet containing virus was re-suspended in EBM-2MV medium. To determine the infectious virus titer, 2.7 x 10^4^ HEK-293 cells were plated per well of a 96 well plate and infected by serial dilution of the virus preparation on the next day. Three days after infection, the number of GFP positive cells was counted and the infectious virus titer was calculated.

For inhibitor treatment, 8 x 10^5^ BJAB-rKSHV.219 cells were plated per well of a 12 well plate and treated with 1μg per ml of anti-human IgM antibody plus the individual inhibitors for the PLCγ (U73122), MAPK (U126) and PI3K/Akt (Ly294002) pathways at their respective concentrations. Seventy two hours later, cells and culture supernatant were collected separately and the expression level of KSHV lytic proteins as well as the activation level of the respective signaling components was analyzed by western blot and infectious virus release was assessed by titrating the culture supernatant on HEK-293 cells.

LECs (between passage 2 and 5) were plated at 5 x 10^5^ cells per well of a 6 well plate and infected with rKSHV.219 virus at a multiplicity of infection (MOI) of 1 by spinoculation (450 x g for 30 minutes at 30°C) in the presence of 10 μg/ml polybrene (H9268; Sigma-Aldrich, Milwaukee, WI, USA). The culture medium was changed after 8 hours. Two days post-infection, the medium was changed to selection medium containing 0.25 μg/ml puromycin and stably infected cells (LEC-rKSHV.219) were maintained under selection for two weeks. For experiments comparing rKSHV.219 infection in LECs and HuARLT2 cells, HuARLT2 cells were infected in parallel in a similar fashion and maintained with 5 μg/ml puromycin and 1 μg/ml doxycycline.

SF-9 cells (DSMZ No.: ACC 125) were grown and maintained in Grace’s insect medium (Gibco, lifetechnologies, Paisley, UK) supplemented with 10% FBS standard quality (PAA Laboratories GmbH, Pasching, Austria) and 100 units/ml of penicillin/streptomycin (CytoGen GmbH, Sinn, Germany) at 28°C. A recombinant baculovirus expressing the KSHV regulator of transcription activation (RTA) was kindly provided by J. Vieira (University of Washington, Seattle WA, USA) and propagated in SF-9 cells. Culture supernatant containing the baculovirus was used in combination with Sodium butyrate (B5887; Sigma-Aldrich, Saint Louis, Mo, USA) for induction of the KSHV lytic cycle in stably infected cells as described before [61].

For the investigation of K15-induced signaling in HEK-293 cells, 2.5 x 10^5^ cells were plated per well of a 12 well plate. On the next day, 1μg of the K15 expression vector pFJ-K15 [42], a kind gift from Jae U. Jung (University of Southern California, Los Angeles, California, USA), or an empty vector control was transfected by using the Fugene 6 transfection reagent (Promega, Madison, WI, USA) following the manufacturer’s protocol. Two days after transfection, cells were washed and lysed; and western blot analysis was performed by using phospho-specific antibodies for the respective signaling components.

### Establishment of KSHV BAC36 stably transfected/ infected cells

A recombinant KSHV genome in the Bacterial artificial chromosome 36 backbone (KSHV-Bac36) [54] was used to construct deletion mutants KSHV-Bac36ΔK1 and KSHV-Bac36ΔK15 by replacing the genes encoding either K1 (nucleotide sequence between 104 and 970) or K15 (nucleotide sequence between 135338 and 136900) with rpsL/neomycin cassette using Red/ET recombination system (Gene Bridges GmbH, Heidelberg, Germany) as described before [48, 94]. The integrity of all three KSHV-Bac36 constructs was verified by whole genome deep sequencing. Briefly, purified Bac DNA was sheared by sonication. To avoid bias by over-amplification, library preparation was performed using the KAPA real-time library preparation kit (KAPA Biosystems, Wilmington, MA, USA). Paired-end 300 bp reads were generated on a MiSeq sequencer by running the v3 chemistry (Illumina, San Diego, CA, USA).

A semi-confluent culture of HEK-293 cells was then transfected with 2 μg of Bac DNA per well of a 6 well plate per each construct (KSHV-Bac36Wt, KSHV-Bac36ΔK1 or KSHV-Bac36ΔK15) using the Fugene 6 transfection reagent. Three days after transfection, cells were re-plated and maintained with a selection medium containing 150 μg/ml of Hygromycin-B (P06-08100; PAN Biotech GmbH, Aidenbach, Germany) until a fully selected polyclonal population for each construct (HEK 293-KSHV-Bac36Wt, HEK 293-KSHV-Bac36ΔK1 or HEK 293-KSHV-Bac36ΔK15) was obtained. 2.5 X 10^5^ stably transfected cells passaged more than 4 times were then plated per well of a 12 well plate. After 24 hours, the KSHV lytic cycle was induced using a reactivation cocktail containing 1mM Sodium butyrate (SB) and SF-9 cell supernatant containing KSHV RTA expressing baculovirus. Forty-eight hours later, the cell culture supernatant was collected and the titer of infectious virus was determined as before (see section cells, viruses and infection), and cells were washed once with 1x PBS and subsequently lysed with lysis buffer for western blot analysis. For experiments involving the PLCγ2-cSH2 domain, 1 μg of the plasmid DNA or its empty vector control was reverse-transfected into 2.5 X 10^5^ HEK 293-KSHV-Bac36Wt cells per well of a 12 well plate during plating, 24 hours after transfection cells were treated with reactivation cocktail as before and subsequent steps were performed similarly. The pTriEx-4 vector expressing the isolated PLCγ2-cSH2 domain was a kind gift from Matilda Katan (University College London, London, UK).

To establish a stably infected HuARLT2 cell line, recombinant viruses were produced from the stably transfected HEK-293-KSHV-BAC36Wt/ΔK1/ΔK15 cells. Briefly, a semi-confluent monolayer of the respective stably transfected cells was treated with a reactivation cocktail containing 1.25 mM Sodium butyrate and SF-9 cell supernatant containing baculovirus expressing KSHV RTA. Cell culture supernatant was collected after 72 hours, filtered through 0.45μm filter and concentrated by ultra-centrifugation at 15,000 rpm for 5 hours in a Type 19 rotor; the pellet containing the respective viruses was re-suspended in EBM-2MV medium. A semi-confluent monolayer of HuARLT2 cells was then infected with the wild type or either of the deletion mutant viruses by spinoculation (450 x g for 30 minutes at 30°C) in the presence of 5μg/ml polybrene. Three days after infection, 100 μg/ml of Hygromycin-B was added and cells were grown and maintained until a fully selected, stably infected, polyclonal population was obtained for each virus (HuARLT2-KSHV-Bac36Wt, HuARLT2-KSHV-Bac36ΔK1 or HuARLT2-KSHV-Bac36ΔK15). 5 X 10^5^ stably infected polyclonal cells passaged more than 4 times were plated per well of a 6 well plate and experiments were performed similar to the stably transfected HEK-293 cells described above.

### Retroviral vector construction, production and transduction of endothelial cells

First, in order to generate the pSF91-PLCγ2-cSH2-IRES-GFP retroviral vector, the PLCγ2-cSH2 domain containing S- and His-tags at the N-terminus was amplified by PCR from the pTriEx-4-PLCγ2-cSH2 expression vector using Fwd: 5’-CCTGCGGCCGCATGGTACACCATCACCACC-3’ and Rev 5’-GTAGCGGCCGCTTATTTACTCGGGGTCACGGGG-3’ primers. The amplified segment was then inserted into the retroviral vector pSF91-IRES-GFP (provided by Christopher Baum; Hannover Medical School, Hannover, Germany) using the *Not I* restriction site. Retroviruses containing the pSF91-PLCγ2-cSH2-IRES-GFP, pSF91-sK15-IRES-GFP or the empty vector control pSF91-IRES-GFP were produced in HEK-293T cells after calcium-phosphate co-transfection of the respective vector constructs together with packaging plasmids pM57DAW (gag/pol) and pRD114 (env) as described before [48]. Lentiviral vector pRRL-PPT-SF-PLCγ2-cSH2 [49] or its empty vector pRRL-PPT-SF-GFP was similarly produced in HEK-293T cells by co-transfection with the helper plasmids pMDLGg/p, pRSV-REV and pMD-G.

A semi-confluent monolayer of endothelial cells (LEC, LEC-rKSHV.219, HuARLT2 or HuARTL2-rKSHV.219) was then transduced with the indicated retroviral/lentiviral vectors by spinoculation (450 x g for 30 minutes at 30°C) in the presence of 5μg/ml of polybrene. After 48 (for K15 overexpression) or 72 hours (for PLCγ2-cSH2), cell culture supernatant and cells were collected for virus titration and western blot analysis, respectively. Transduction efficiency was estimated by infecting KSHV-negative parental cells (LEC, HuARLT2) and scoring the number of GFP positive cells.

### SiRNA transfection

Transfection of small interfering RNA (siRNA) in BJAB-rKSHV.219 and HuARLT2-rKSHV cells was performed by using the Neon transfection system (Invitrogen, Carlsbad, CA, USA) according to the manufacturer’s instruction. 150 pmol of each individual siRNA was microporated into 4 x 10^5^ BJAB-rKSHV.219 or 1 x 10^5^ HuARLT2-rKSHV cells and the KSHV lytic cycle was induced the next day by using anti-human IgM antibody or a cocktail of KSHV RTA and Sodium butyrate. Cell culture supernatant and cells were separately collected after 48 hours of lytic induction and infectious virus titer as well as the expression level of KSHV lytic proteins was analyzed subsequently.

LECs or LEC-rKSHV.219 cells were transiently transfected with individual siRNA complexed with Lipofectamin RNAiMAX reagent (Invitrogen, life technologies, Carlsbad, CA, USA) as per the manufacturer’s recommendation. Briefly, 150 pmol of each individual siRNA and 6 μl of Lipofectamin RNAiMAX reagent were each diluted in 150 μl of low serum Opti-MEM (Gibco, lifetechnologies, Paisley, UK) media and combined. The siRNA: transfection reagent complex was then incubated for 20 minutes at room temperature and applied on to a semi-confluent monolayer of cells in a well of a 6 well plate. Cell culture supernatant and cells were collected 72 hours after transfection for virus titration and western blot analysis respectively. Control/ non-targeting scrambled (Scr) siRNA pool #2 D-001206-14-20, siRNA against KSHV K15 targeting exon 8 #1 (CAACCACCUUGGCAAUAAU), siRNA against KSHV K15 targeting exon 8 #2 (CAGGCUUGGUCAUGGGUUA), siRNA against KSHV K1 (GUCACUUGUGGUCAGCAUG) and siRNA against KSHV ORF 26 (CCAUUGUGCUCGAAUCCAA) were all purchased from Dharmacon, Thermo Scientific.

### Western blot analysis

Cells were lysed with 1x SDS sample buffer (62.5 mM Tris-HCl pH 6.8, 2% (W/V) SDS, 10% (V/V) Glycerol, 50 mM DTT, 0.01% (V/V) β-mercaptoethanol and 0.01% (W/V) bromophenol blue) and centrifuged at 20,000 x g for 10 minutes at 4°C. Cleared total cellular lysate was then separated by SDS-PAGE (Note: for the analysis of KSHV K15 protein, samples were not boiled prior to SDS-PAGE) and transferred onto 0.45 μm nitrocellulose membranes (Amersham, GE healthcare Europe GmbH, Freiburg, Germany). Membranes were then blocked with 5% (W/V) non-fat milk (Carl Roth GmbH+Co.KG, Karlsruhe, Germany) or IgG free albumin (Carl Roth GmbH&Co.KG, Karlsruhe, Germany) for 1 hour and probed with an appropriate primary antibody in blocking solution overnight, at 4°C. The next day, membranes were washed three times and incubated with the corresponding horseradish peroxidase (HRP)-conjugated secondary antibodies for an hour at room temperature (RT). After a subsequent wash, signal was developed using a standard enhanced chemiluminescence (ECL) kit (#34096; Thermo Scientific, Rockford, IL, USA). Primary and secondary antibodies used for western blot are listed above (section antibodies).

### Membrane flotation assay

7x 10^7^ 293-KSHV-BAC36Wt or HuARTL2-rKSHV cells, with or without induction of the KSHV lytic cycle as described above, were collected, washed once with 1xPBS and lysed in 1 ml of ice-cold TNE buffer (10mM Tris-HCl pH 7.4, 150mM NaCl, 5mM EDTA) containing 1% Triton X-100 and protease inhibitors and incubated on ice for 30 minutes. Cellular lysates were then homogenized by passing through a 200 μl pipet tip 10 times and centrifuged at 900 x g at 4°C for 10 minutes. Cleared supernatant was then mixed with 1 ml of 85% sucrose in TNE buffer in a 1:1 ratio and pipetted at the bottom of a 14 X 95 mm ultracentrifuge tube (Beckman Coulter Inc., Brea, CA, USA), overlaid with first 6 ml of 35% and then 3.5 ml of 5% sucrose solutions in TNE buffer and subjected to ultracentrifugation at 200,000 x g for 24 hours at 4°C in an SW40 rotor (Beckman). Twelve, fractions of 1 ml were then collected starting from the top of the gradient and analyzed for the desired proteins by western blot.

### Immunofluorescence assay

For overexpression experiments, Hela CNX cells were plated on sterile 20 x 20 mm cover slips (1 x 10^5^ cells per well of a 6 well plate). On the next day, cells were transfected with 2 μg each of the respective plasmid DNA using the Fugene 6 transfection reagent. Forty-eight hours after transfection, cells were washed with 1x PBS, fixed with 4% paraformaldehyde (PFA) in PBS for 20 minutes at RT and quenched with 125 mM glycine for 10 minutes at RT. After 3 washes in 1xPBS cells were permeablized with 0.2% Triton X-100 for 10 minutes at RT, blocked with 10% FBS in PBS for an hour at 37°C and incubated with the respective primary antibody in blocking solution for an hour at 37°C. After another 3 washes, cells were incubated with the corresponding fluorescently labeled secondary antibody for an hour at 37°C. Plasmid constructs used include pFJ-K15P/M (encoding either K15P or M). For staining endogenous proteins in infected cells, 2 x 10^5^ HuARLT2-rKSHV cells were plated on cover slips as before and the KSHV lytic cycle was induced the next day using RTA and SB. Forty-eight hours after lytic induction, cells were washed in 1xPBS and fixed with 100% ice cold methanol at -20°C. Coverslips were then washed thoroughly with 1xPBS, blocked with 10% FBS in PBS for an hour at 37°C and stained with the respective primary antibodies and secondary antibodies as described above. For staining in LEC-rKSHV cells, samples were processed similar to HuARLT2-rKSHV cells but without induction of the lytic cycle.

Images were acquired with a Leica TCS SP2 AOBS confocal microscope (Leica Microsystems, Wetzlar, Germany) or with a Zeiss Axio Observer.Z1 epi-fluorescent microscope (Carl Zeiss Iberia, Madrid, Spain).

### K15 staining in KS tissue

To embed HuARLT2 and HuRLT2-rKSHV cells in paraffin we used a previously described protocol [102]. Briefly, cells were harvested, pelleted by centrifugation and fixed in 4% PFA for 20 minutes at room temperature. After 3 washes in 1XPBS the cell pellets were re-suspended in 500 μl of 1.5% (W/V) melted (50°C) agarose basic (Aplichem GmbH, Darmstadt, Germany) in PBS and quickly transferred to a capped inverted 2ml Eppendorf tube. The agar-cell suspension was left on ice to solidify and gently extracted by opening the tube cup. The agar cylinder was then embedded in paraffin after processing as for histopathology specimens. The HuARLT2 and HuARLT2-rKSHV cell blocks were then used for testing the suitability of the rat anti-K15 mAb (clone 18E5) for K15 staining in paraffin embedded tissue and as a negative and positive control during KS tissue staining.

Paraffin embedded cell blocks and biopsy samples obtained from HIV positive KS patients were sectioned into 5μm thick slices and deparaffinized in Roti-Histol (Carl Roth GmbH, Karlsruhe, Germany). After rehydration in descending ethanol dilution series, antigen retrieval was performed using a citrate-based antigen unmasking solution (H-3300, Vector Laboratories; 15 minutes, 100°C). Endogenous peroxidases were blocked with 3% H_2_O_2_/H_2_O for 10 minutes and staining was performed using the rat anti-K15 mAb (clone 18E5) [49] as primary antibody followed by a biotinylated goat anti-rat IgG (1:250, 111065033; Dianova) secondary antibody. The signal of the rat anti-K15 mAb was amplified using the Tyramide Signal Amplification (TSA) system (NEL702001KT; Perkin Elmer) and nuclei were counterstained with 4',6-diamidino-2-phenylindole (DAPI).

Images were acquired using a Leica DM6000 microscope with Leica DFC350FX digital camera and contrast and brightness was adjusted afterwards in Adobe Photoshop CS4.

## Supporting information

S1 Fig Sequence coverage after deep sequencing of KSHV-BAC36 Wt/ΔK1/ΔK15 constructs. (TIF)

S2 Fig Depleting PLCγ1 mRNA from KSHV stably infected cells compromises its ability to undergo lytic reactivation. HuARLT2-rKSHV cells were microporated with either a control siRNA (Scr) or siRNA against PLCγ1 and the lytic cycle was induced 24 hours later. Forty eight hours after lytic induction, **(A)** images for GFP and RFP expression were acquired and **(B)** the number of RFP positive cells from nine fields was quantified; **(C)** cells were then lysed and the expression level of the indicated lytic viral proteins was assessed by western blot. Results are representative of two independent experiments. Bar graphs **(B)** represent the means ± SD of nine fields.
(TIF)

S3 Fig The isolated PLCγ2-cSH2 domain blocks KSHV reactivation. HuARLT2-rKSHV cells were transduced with a lentivirus vector expressing the PLCγ2-cSH2 domain or an empty vector control and the KSHV lytic cycle was induced 24 hours later. Forty eight hours after induction of the lytic cycle, images were taken for GFP and RFP expression **(A)**, cells were lysed and **(B)** expression level of the indicated viral proteins was analyzed by western blot as well as **(C)** KSHV infectious virus titer in the cell culture supernatant was determined by infecting HEK-293 cells and counting GFP expressing cells. Experiments were performed two or more times. Bar graphs in **(C)** represent the means ± SD of 2 independent experiments.
(TIF)

S4 Fig KSHV lytic reactivation in HuARLT2-rKSHV cells. 5 x 10^5^ HuARLT2-rKSHV cells were plated and the KSHV lytic cycle was induced 24 hours later using a cocktail of RTA and SB. After 48 hours of induction, images were taken for GFP and RFP expression from cells with or without induction of the lytic cycle.
(TIF)

S5 Fig The rat anti-K15 mAb (clone number 18E5) detects a conserved motif surrounding an SH2 binding site in both K15M and K15P proteins. **(A)** and **(B)** An array of 44 overlapping peptides spotted on microscope glass slides were stained with a rat anti-K15 antibody 18E5 (used for IF and IHC) or number 10A6 (used for western blot), followed by a Cy3-conjugated anti-rat IgG (green), a Cy5-conjugated streptavidin (red) was used to bind to biotin spots marking the border of the peptide array spots. Both antibodies 18E5 and 10A6 recognized the sequence PTDDLYEEVLFP surrounding the SH2 domain-binding site at the c-terminal of the K15 cytoplasmic tail. **(C)** Hela-CNX cells transfected with K15P or K15M were stained with the rat anti-K15 mAb 18E5 followed by a Cy3-conjugated anti-rat IgG (red) secondary antibody and cell nuclei were counter stained with DAPI. As an additional specificity control, the primary antibody was omitted in the images in the bottom row.
(TIF)
