# Supplementary figures and images for: The Kaposi's sarcoma-associated herpesvirus (KSHV) non-structural membrane protein K15 is required for viral lytic replication and may represent a therapeutic target
Source: PLoS Pathog. 2017 Sep 22;13(9):e1006639. doi: 10.1371/journal.ppat.1006639 (PMC5627962; doi:10.1371/journal.ppat.1006639)

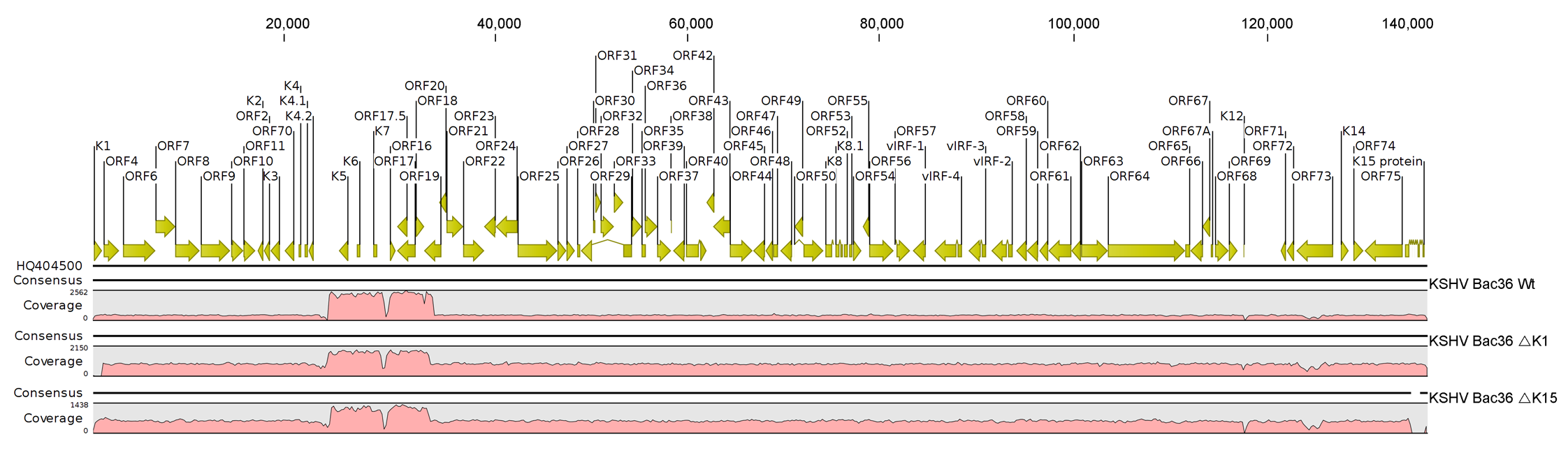

Supplement: S1 Fig — (TIF) [file ppat.1006639.s001.tif]

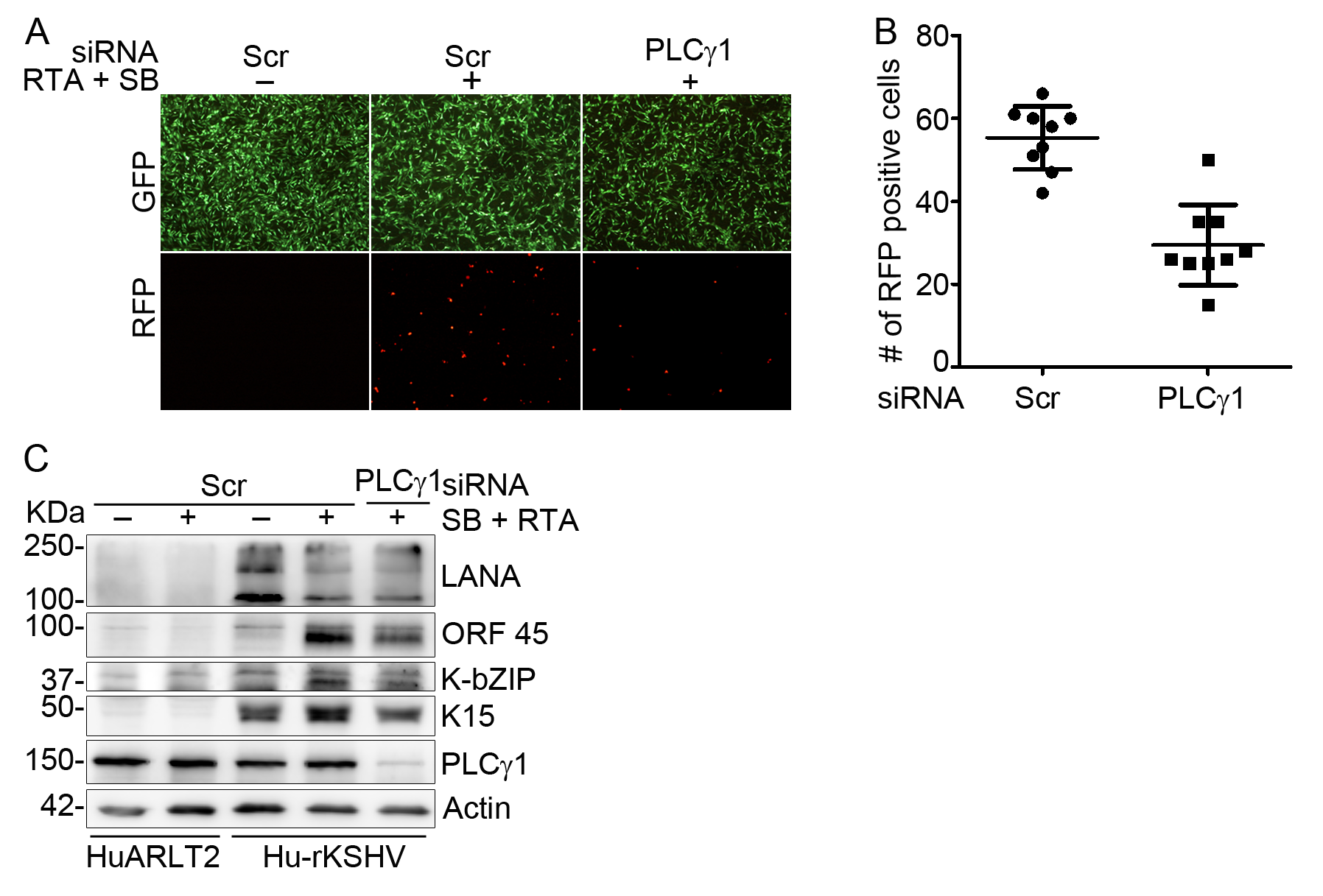

Supplement: S2 Fig — HuARLT2-rKSHV cells were microporated with either a control siRNA (Scr) or siRNA against PLCγ1 and the lytic cycle was induced 24 hours later. Forty eight hours after lytic induction, (A) images for GFP and RFP expression were acquired and (B) the number of RFP positive cells from nine fields was quantified; (C) cells were then lysed and the expression level of the indicated lytic viral proteins was assessed by western blot. Results are representative of two independent experiments. Bar graphs (B) represent the means ± SD of nine fields. (TIF) [file ppat.1006639.s002.tif]

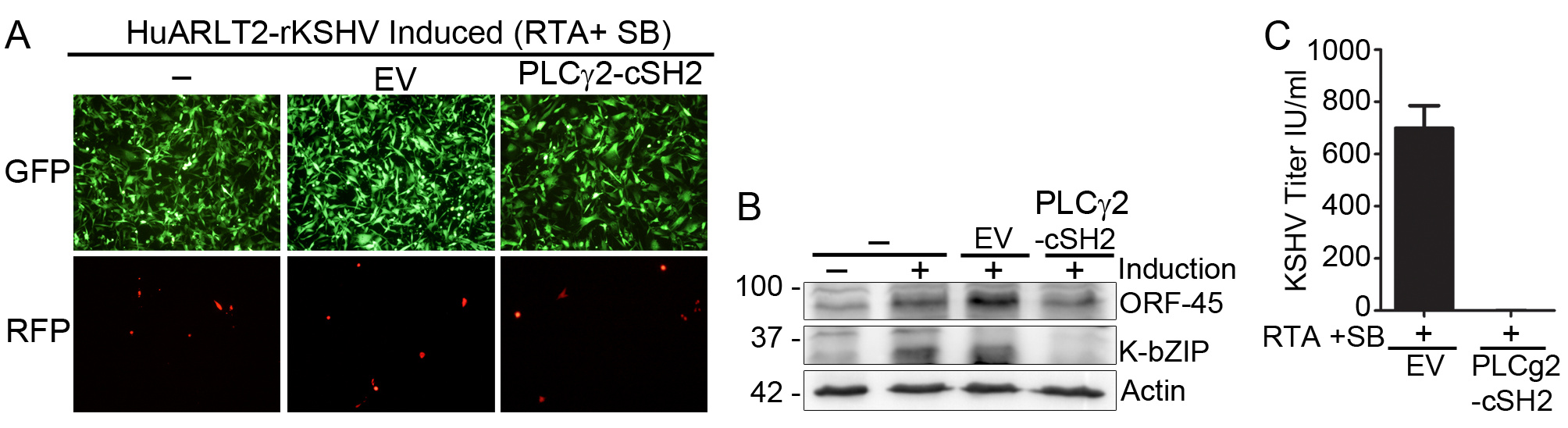

Supplement: S3 Fig — HuARLT2-rKSHV cells were transduced with a lentivirus vector expressing the PLCγ2-cSH2 domain or an empty vector control and the KSHV lytic cycle was induced 24 hours later. Forty eight hours after induction of the lytic cycle, images were taken for GFP and RFP expression (A), cells were lysed and (B) expression level of the indicated viral proteins was analyzed by western blot as well as (C) KSHV infectious virus titer in the cell culture supernatant was determined by infecting HEK-293 cells and counting GFP expressing cells. Experiments were performed two or more times. Bar graphs in (C) represent the means ± SD of 2 independent experiments. (TIF) [file ppat.1006639.s003.tif]

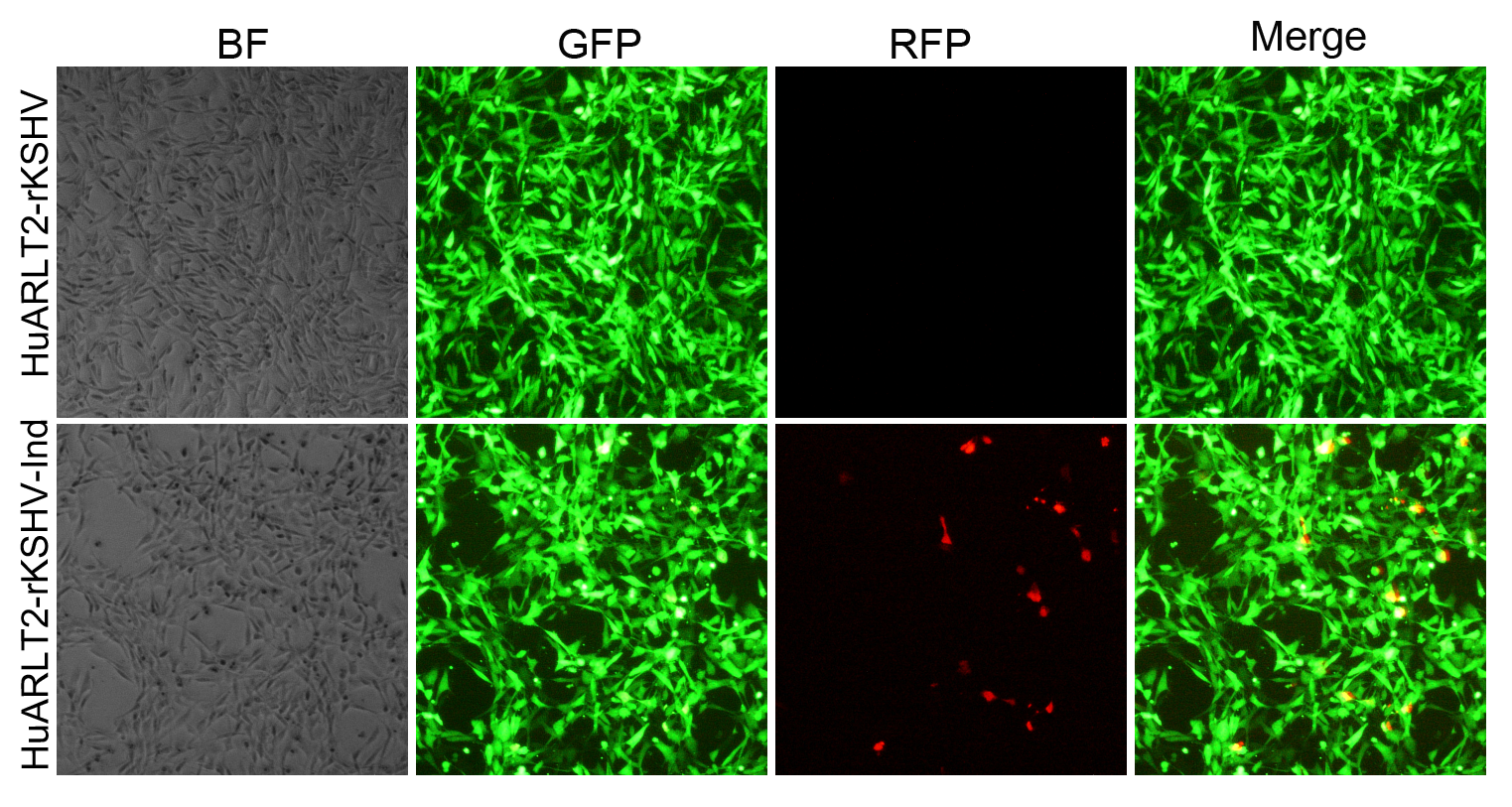

Supplement: S4 Fig — 5 x 10^5^ HuARLT2-rKSHV cells were plated and the KSHV lytic cycle was induced 24 hours later using a cocktail of RTA and SB. After 48 hours of induction, images were taken for GFP and RFP expression from cells with or without induction of the lytic cycle. (TIF) [file ppat.1006639.s004.tif]

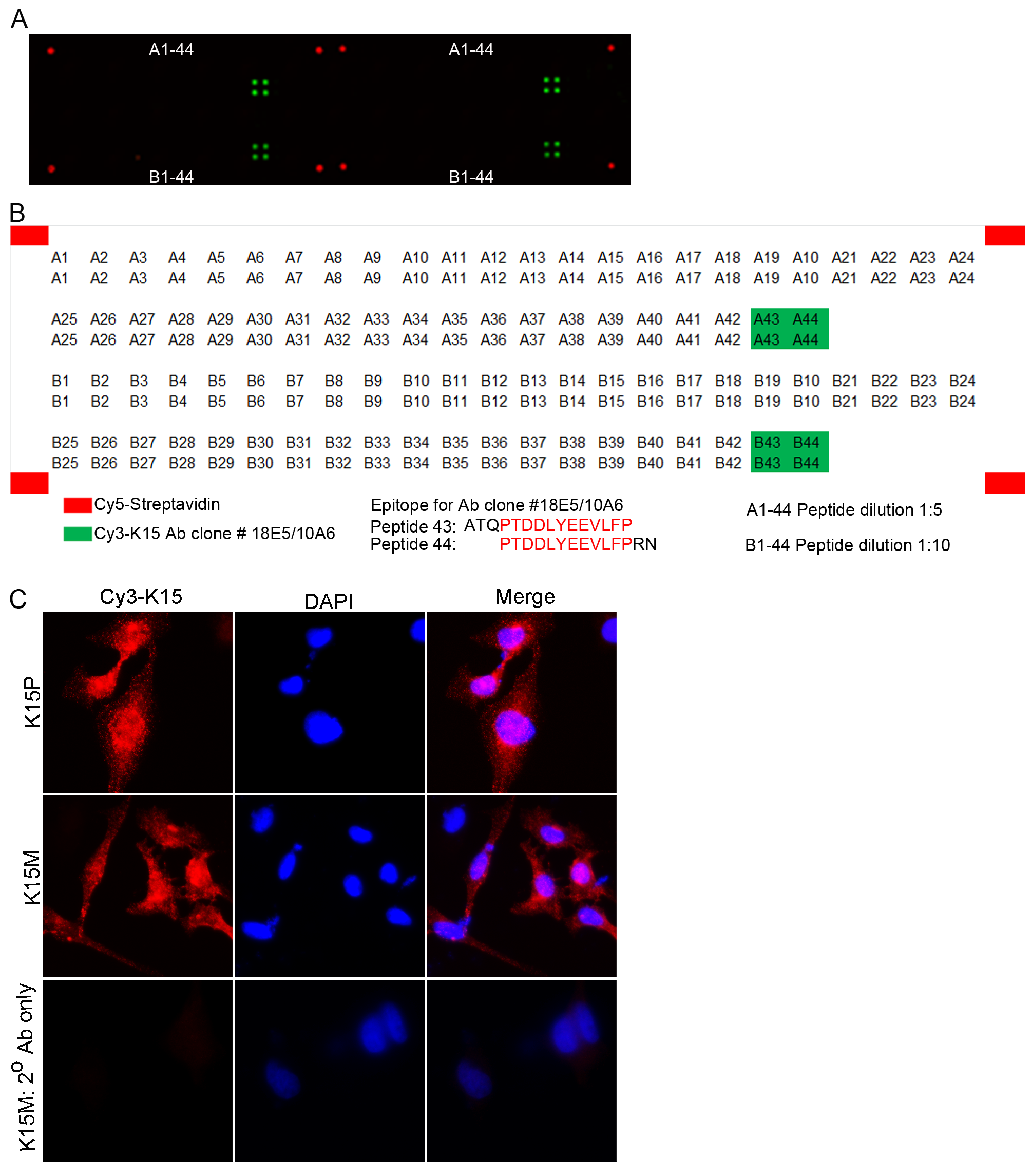

Supplement: S5 Fig — (A) and (B) An array of 44 overlapping peptides spotted on microscope glass slides were stained with a rat anti-K15 antibody 18E5 (used for IF and IHC) or number 10A6 (used for western blot), followed by a Cy3-conjugated anti-rat IgG (green), a Cy5-conjugated streptavidin (red) was used to bind to biotin spots marking the border of the peptide array spots. Both antibodies 18E5 and 10A6 recognized the sequence PTDDLYEEVLFP surrounding the SH2 domain-binding site at the c-terminal of the K15 cytoplasmic tail. (C) Hela-CNX cells transfected with K15P or K15M were stained with the rat anti-K15 mAb 18E5 followed by a Cy3-conjugated anti-rat IgG (red) secondary antibody and cell nuclei were counter stained with DAPI. As an additional specificity control, the primary antibody was omitted in the images in the bottom row. (TIF) [file ppat.1006639.s005.tif]
